# Non-Isocyanate Aliphatic–Aromatic Poly(carbonate-urethane)s—An Insight into Transurethanization Reactions and Structure–Property Relationships

**DOI:** 10.3390/ijms231910999

**Published:** 2022-09-20

**Authors:** Dominik Wołosz

**Affiliations:** Faculty of Chemistry, Warsaw University of Technology, Noakowskiego 3, 00-664 Warsaw, Poland; dominik.wolosz.dokt@pw.edu.pl

**Keywords:** non-isocyanate polyurethanes, aromatic polyurethanes, transurethanization, polycondensation, polycarbonates

## Abstract

This study reveals insights into the transurethanization reactions leading to the aliphatic–aromatic non-isocyanate poly(carbonate-urethane)s (NIPCUs) and their structure–property relationships. The crucial impact of the alkyl chain length in 4,4′-diphenylmethylene bis(hydroxyalkyl carbamate) (BHAC) on the process of transurethanization reactions was proved. The strong susceptibility of hydroxyethyl- and hydroxybutyl carbamate moieties to the back-biting side reactions was observed due to the formation of thermodynamically stable cyclic products and urea bonds in the BHACs and NIPCUs. When longer alkyl chains (hydroxypentyl-, hydroxyhexyl-, or hydroxydecyl carbamate) were introduced into the BHAC structure, it was not prone to the back-biting side reaction. Both ^1^H and ^13^C NMR, as well as FT-IR spectroscopies, confirmed the presence of carbonate and urethane (and urea for some of the samples) bonds in the NIPCUs, as well as proved the lack of allophanate and ether groups. The increase in the alkyl chain length (from 5 to 10 carbon atoms) between urethane groups in the NIPCU hard segments resulted in the increase in the elongation at break and crystalline phase content, as well as the decrease in the *T_g_*, tensile strength, and hardness. Moreover, the obtained NIPCUs exhibited exceptional mechanical properties (e.g., tensile strength of 40 MPa and elongation at break of 130%).

## 1. Introduction

Non-isocyanate polyurethanes (NIPUs) have emerged with a huge potential to replace typical polyurethanes (PUs) due to their relatively easy synthetic procedure and reduction in volatile organic solvents and diisocyanates. Negative aspects of the application of diisocyanates for PU preparation involve the phosgene-based synthesis, moisture sensitivity, and their constantly increasing price [1,2,3,4]. In contrast, the NIPUs’ precursors are not phosgene-based and are not moisture sensitive. Furthermore, they can be obtained from carbon dioxide—a greenhouse gas that is easily accessible [5,6].

The typical synthesis of PUs poses some issues. One of them is the strong relationship between the stoichiometric ratio of reactants and the molar mass of the final product. Moreover, side products containing biuret and allophanate bonds can be formed depending on the temperature, type of catalyst, and presence of even a trace of water [7]. However, they are not the only issues. Diisocyanate vapors and their residuals in the final product can cause some health problems during manufacturing and use [8], which is why the transition toward a more sustainable synthesis method of PUs is desired. This is evidenced by the extensive range of PU applications (e.g., foams, coatings, adhesives, and varnishes) [9,10,11,12,13,14] and their constantly growing global market, which is valued at around EUR 53 billion [15].

One of the representative classes of PUs is poly(carbonate-urethane)s (PCUs), which are obtained from oligocarbonate diol (OCD) as a soft segment precursor. Recently, PCUs have been gaining interest due to their exceptional oxidative and hydrolytic resistance compared to poly(ether-urethane)s and poly(ester-urethane)s [16]. Interestingly, the introduction of carbonate groups into the PU structure also improves their mechanical and thermal properties due to the greater density of hydrogen bonds and bigger influence on the microphase separation [17,18].

In contrast with the typical synthetic pathway, NIPUs can be obtained via the transurethane polycondensation method with the use of stable, moisture-insensitive reagents (carbamates, α,ω-diols, and/or oligodiols). Then, the molar mass of the NIPUs is independent of the stoichiometric ratio between the reactants, hence it can be adjusted by the number of low-molar-mass products distilled off the reacting mass [19].

In this method, the physicochemical properties of NIPUs can be controlled by an appropriate selection of hard (carbamates) and soft segment precursors (oligoether diols, oligoester diols, or OCDs) [20,21,22,23,24,25,26,27]. The exemplary methods for increasing the rigidity of the NIPUs are the increase in the hard segment contents or the application of aromatic carbamates [19].

Carbamates are commonly synthesized via the methoxycarbonylation of aliphatic/aromatic diamines with dimethyl carbonate [28,29,30] or aminolysis of ethylene carbonate using aliphatic diamines [6,22,31,32]. The products of these reactions are bis(methyl carbamate)s (BMC) or bis(2-hydroxyalkyl carbamate)s, respectively. Due to the lowered electrophilicity of the aromatic diamines compared to the aliphatic ones, they can be used only in the case of a reaction with dimethyl carbonates at an elevated temperature. When they react with ethylene carbonate, they are inactive [33] or lead to the formation of oligoether diols [34] and cyclic carbamates [35], depending on the reaction conditions. This is why the aromatic diamine-based NIPUs can only be obtained based on dimethyl carbonate derivatives.

The first report concerning the non-solvent transurethane polycondensation of aliphatic BMC with α,ω-diols (C8 and C18) toward aliphatic non-isocyanate [n,m] PUs was revealed by Meier et al. [28] A similar synthetic route toward aliphatic non-isocyanate poly(carbonate-urethane)s (NIPCUs) was reported by Dongdong and Hengshui [36]. The study referred to the direct transurethane polycondensation of BMC with OCDs. However, Shen et al. noticed that the direct reaction of BMC with a high-molar-mass diol (OCD) led to covalently crosslinked side products due to the reaction of the secondary amide with the methoxycarbonyl groups [37]. To solve this problem, they first obtained a NIPCU hard segment precursor via the reaction of aliphatic BMC with 1,6-hexanediol and consecutive transurethane polycondensation. Then, the obtained product was subjected to transurethane polycondensation, with crystalline OCD yielding non-crosslinked aliphatic NIPCU. Similarly, our research group has studied the reactions of different aliphatic (C4, C6, and C8) and aromatic (4,4′-diphenylmethylene or 2,4-toluilene) BMCs with asymmetric α,ω-diol (1,5-pentanediol) [19]. In contrast to the study by Shen et al., we have carried out transurethanization with a molar excess of α,ω-diol under the flow of inert gas to facilitate the diffusion of the methanol from the reacting mass. Thanks to this operation, it was possible to obtain monomeric aliphatic or aliphatic–aromatic bis(5-hydroxypentyl carbamate) diols. In the next step, the obtained products were subjected to transurethane polycondensation with amorphous oligo(pentamethylene-*co*-hexamethylene carbonate) diol, yielding non-crosslinked and urea-free NIPCUs.

The type of α,ω-diol, used during transurethanization with BMC, affects the reaction process, determines the possible occurrence of side reactions, and defines the structure of hard segments and properties of the NIPUs [6,19,32,38]. On one hand, the application of shorter α,ω-diol (e.g., 1,2-ethanediol) facilitates the further diffusion of low-molar-mass products during transurethane polycondensation due to its lower boiling point. However, the presence of 2-hydroethyl carbamate moieties accelerates the formation of urea groups via back-biting side reactions [6,31,32]. The increase in the chain length of α,ω-diol decreases the tendency to form urea groups [38].

In this study, the influence of α,ω-diol chain lengths (C2, C4, C5, C6, and C10) on the process of transurethanization with 4,4′-diphenylmethylene BMC and consecutive transurethane polycondensation with crystalline oligo(hexamethylene carbonate) diol was revealed. Moreover, the distillates after transurethanization and transurethane polycondensation were analyzed using ^1^H NMR spectroscopy to study the possibility of the formation of side groups (urea groups). An additional aim was to determine the influence of the hydrocarbon chain length in the hydroxyalkyl carbamate groups of aliphatic–aromatic NIPCUs on their mechanical and thermal properties. This work is the first observation of the structure–property relationships and side reactions that occur during the synthesis of aliphatic–aromatic NIPCUs.

## 2. Results and Discussion

**Scheme 1:**
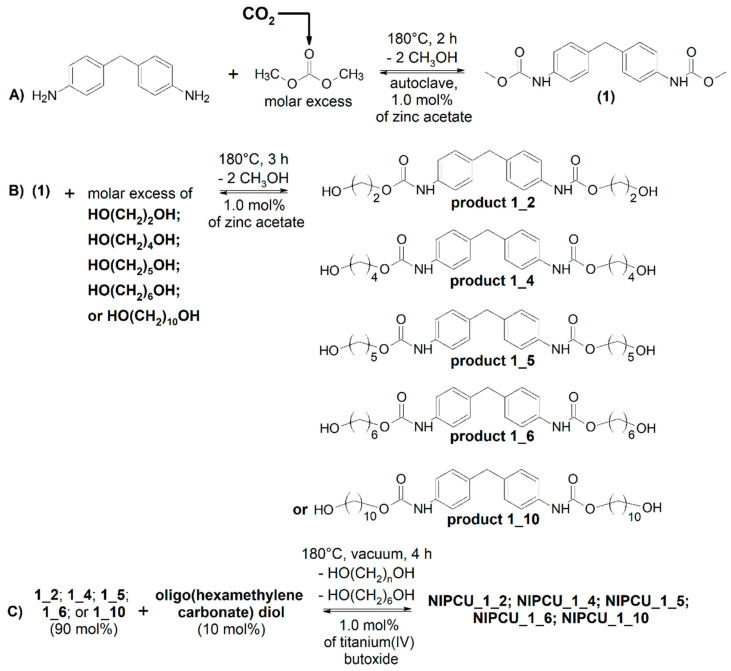
The phosgene- and diisocyanate-free synthesis pathways to aliphatic–aromatic NIPCUs with different structures of hard segments: (**A**)—methoxycarbonylation of the MDA, (**B**)—transurethanization of the BMC with α,ω-diol (C2-C10), and (**C**)—transurethane polycondensation of the aliphatic–aromatic BHAC with crystalline oligo(hexamethylene carbonate) diol (number-average molar mass of 2000 g∙mol^−1^) under a vacuum.

Scheme 1 presents the synthetic procedure of the aliphatic–aromatic NIPCUs based on dimethyl carbonate as a green substitute for phosgene. It consists of three main steps: (A) methoxycarbonylation of the 4,4′-diaminodiphenylmethane (MDA), (B) transurethanization of the BMC with α,ω-diol (C2-C10), and (C) transurethane polycondensation of the aliphatic–aromatic bis(hydroxyalkyl carbamate) (BHAC) with crystalline oligo(hexamethylene carbonate) diol (number-average molar mass of 2000 g∙mol^−1^) under a vacuum.

After step A, 4,4′-diphenylmethylene BMC (1) was obtained as a white powder with a high yield (94%). The spectroscopic data (^1^H and ^13^C NMR, as well as FT-IR spectroscopies) and the melting point of 1 were in agreement with the literature data [19,30].

During the second step (B), the precursor of the NIPCU hard segments (aliphatic–aromatic BHAC) differing in the alkylene chain length (C2, C4, C5, C6, or C10) in the terminal groups was obtained. It was crucial to obtain bifunctional hydroxyl-terminated molecules that would be active during transurethane polycondensation with OCD afterward.

The obtained aliphatic–aromatic BHACs (1_2; 1_4; 1_5; 1_6; or 1_10) were subjected to transurethane polycondensation (step C) with 10 mol% of oligo(hexamethylene carbonate) diol yielding aliphatic–aromatic NIPCU varying in the structure of hard segments (NIPCU_1_2; NIPCU_1_4; NIPCU_1_5; NIPCU_1_6; NIPCU_1_10). The synthesis was carried out under a high vacuum (about 0.02 mbar) to shift the reaction equilibria toward the high-molar-mass NIPCU. During this step, OCD was used as the NIPCU soft segment precursor.

### 2.1. Transurethanization of Arylene BMC with α,ω-diol (C2, C4, C5, C6, or C10)

To provide insight into the structure–property relationships of the aliphatic–aromatic NIPCUs, transurethanization between the 4,4′-diphenylmethylene BMC and α,ω-diol (C2–C10) was used to obtain the diverse BHAC (precursor of the NIPCU hard segments). Moreover, the application of different chain-length α,ω-diols enabled the study of the tendency to the formation of side products in the case of both transurethanization and transurethane polycondensation.

**Figure 1:**
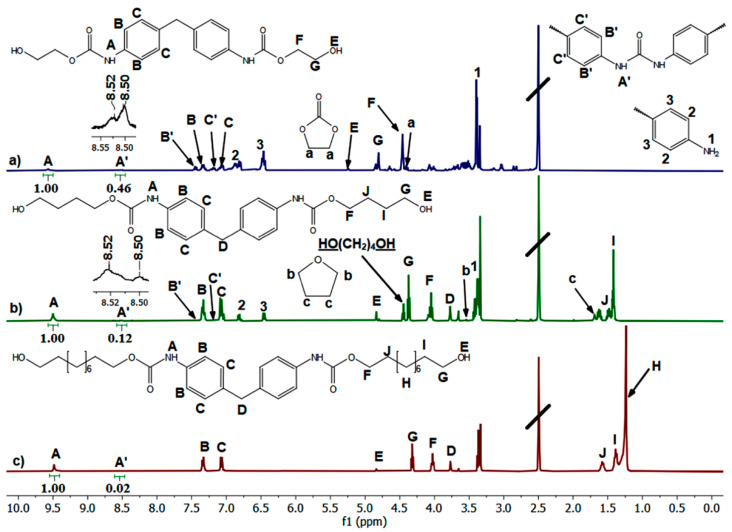
The structure comparison of the BHACs obtained from C2, C4, and C10 α, ω-diol: product 1_2 (**a**), 1_4 (**b**), and 1_10 (**c**).

The process of the transurethanization of 4,4′-diphenylmethylene BMC with α,ω-diol (reaction scheme is shown in Scheme 1B) was followed based on the integral intensity of the methoxycarbonyl (-NHC(O)OCH_3_) groups’ signal (about 3.60 ppm) using ^1^H NMR spectroscopy. The -NHC(O)OCH_3_ groups were entirely exchanged after 3 h of the reaction at 180 °C. Then, the new characteristic signals from the OH, -CH_2_OH, and -NHC(O)OCH_2_ groups were observed at about 4.83 ppm, 4.37 ppm, and 4.00 ppm in the ^1^H NMR spectra, confirming that bifunctional hydroxyl-terminated molecules were obtained (Figure 1). Due to the presence of a short hydrocarbon chain in the 1_2 product, the corresponding signals were noticed at higher chemical shifts of about 5.23 ppm, 4.80 ppm, and 4.46 ppm. The final products were solids of good solubility in common solvents.

**Figure 2:**
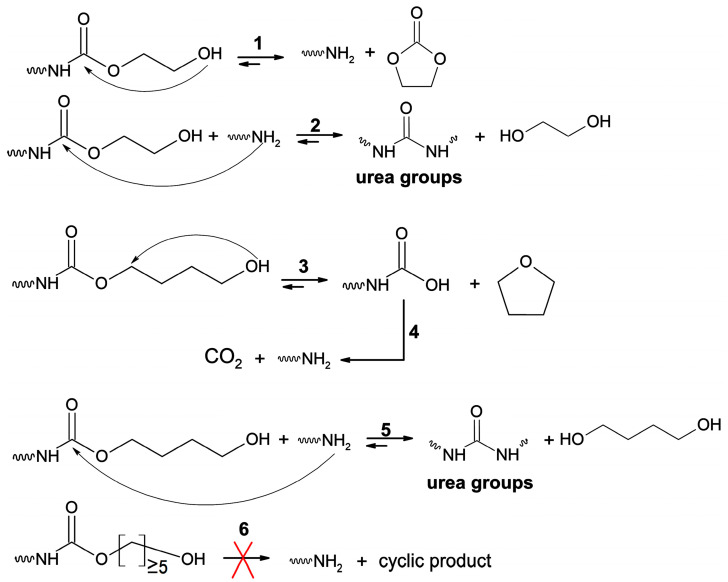
The dependence of the hydrocarbon chain length in the terminal groups of BHACs on the tendency to the formation of urea groups via the back-biting side reactions.

The type of α, ω-diol used during the transurethanization with BMC had a high impact on the occurrence of side reactions. The application of short 1,2-ethanediol and 1,4-butanediol led to the formation of a high concentration of urea groups (-NHC(O)NH-) observed at about 8.50 ppm [39] in the ^1^H NMR spectra (see Figure 1). The concentration of urea bonds was calculated in relation to the urethane groups (9.50 ppm, -NHC(O)O-) based on the ^1^H NMR spectra and equaled 18.80 mol% and 5.48 mol% for the 1_2 and 1_4 products, respectively. Moreover, the spectra proved the presence of MDA and cyclic products (ethylene carbonate or tetrahydrofuran in the case of 1,2-ethanediol or 1,4-butanediol usage, respectively) in the post-reaction mixtures (see Figure 1), demonstrating that the intramolecular side reactions (back-biting) took place. The mechanisms of the back-biting reactions differed depending on the length of the hydrocarbon chain in the BHAC. In the case of bis(2-hydroxyethyl carbamate)s, the terminal OH group attacked the nearest carbonyl carbon atom yielding the thermodynamically stable 5-membered ring of ethylene carbonate and free amine (reaction 1, Figure 2). This consequently caused the nucleophilic attack of the amine group on the carbamate group’s carbonyl carbon atom leading to the formation of urea groups (reaction 2, Figure 2) [6,31].

Interestingly, when longer bis(4-hydroxybutyl carbamate)s were used, the corresponding 7-membered cyclic carbonate was not observed in the post-reaction mixture and distillate. This proved that the OH terminal group preferred the attack on the alkyl carbon atom leading to the formation of a thermodynamically stable product (5-membered ring of tetrahydrofuran) (reaction 3, Figure 2). Another product of this reaction was an unstable carbamic acid derivative that generated the free amine group and consequently urea moieties (reactions 4 and 5, Figure 2). Importantly, the tetrahydrofuran cannot be formed via the cyclization of 1,4-butanediol, since the urea groups would not be present in the final product. The application of α,ω-diol with at least five carbon atoms during transurethanization led to the almost entire limitation of the back-biting side reaction occurrence (Figures S3–S5 and Figure 1, and reaction 6, Figure 2) since the concentrations of urea groups in the case of the 1_5, 1_6, and 1_10 products equaled only 1–2 mol%. These reactions were not thermodynamically preferred due to the presence of long hydrocarbon chains.

**Figure 3:**
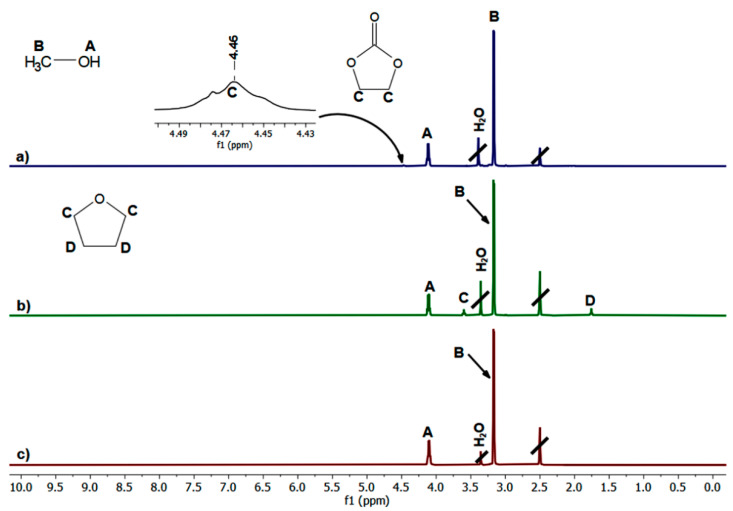
The composition comparison of BHAC products 1_2 (**a**), 1_4 (**b**), and 1_10 (**c**)’s distillate.

The occurrence of the back-biting side reactions was confirmed by the structure analysis of the distillate after transurethanization using ^1^H NMR spectroscopy. In the case of 1,2-ethanediol and 1,4-butanediol used during transurethanization, ethylene carbonate (1.49 mol%) and tetrahydrofuran (4.65 mol%) were observed along with methanol in the distillates, respectively. When longer α,ω-diol with at least five carbon atoms was used, only the characteristic signals of the methanol were noticed (Figure 3 and Figures S6–S10).

### 2.2. Transurethane Polycondensation toward the Aliphatic–Aromatic NIPCUs

**Scheme 2:**
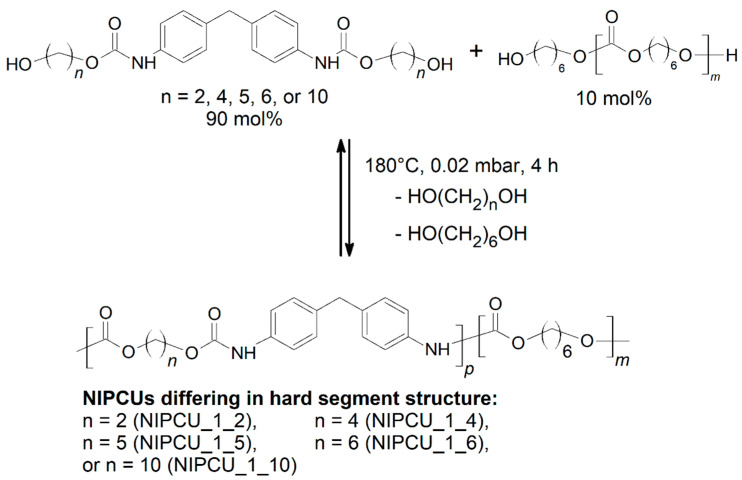
The non-solvent transurethane polycondensation between the proper BHAC and OCD leading to the aliphatic–aromatic NIPCU differing in the structure of hard segments.

The BHACs (1_2, 1_4, 1_5, 1_6, or 1_10) obtained after transurethanization was applied as the NIPCU hard segment precursor during the non-solvent transurethane polycondensation with 10 mol% of crystalline oligo(hexamethylene carbonate) diol of a 2000 g·mol^−1^ number-average molar mass (NIPCU soft segment precursor). The syntheses were performed at an elevated temperature (180 °C) under a vacuum (0.02 mbar) to facilitate the diffusion of the low-molar-mass products from the reacting mass and to shift the reaction equilibria toward the formation of a high-molar-mass NIPCU. The syntheses were finished when the low-molar-mass products stopped distilling (about 4 h). The transurethane polycondensation scheme is presented in Scheme 2. The products NIPCU_1_2 and NIPCU_1_4 were brittle, whereas NIPCU_1_5, NIPCU_6, and NIPCU_1_10 were very tough and stiff. All of them exhibited quite good solubility in dimethyl sulfoxide.

The application of the BHACs with different lengths of hydrocarbon chains (C2-C10) in the terminal hydroxyalkyl carbamate groups during the transurethane polycondensation with OCD allowed for obtaining the diverse aliphatic–aromatic NIPCU with C2-C10-based hard segments. This is why the influence of the hydrocarbon chain length in the NIPCU hard segments on the structure, as well as the thermal and mechanical properties of the final products, should be explored.

The structures of the obtained NIPCUs were studied using ^1^H and ^13^C NMR as well as FT-IR spectroscopies.

**Figure 4:**
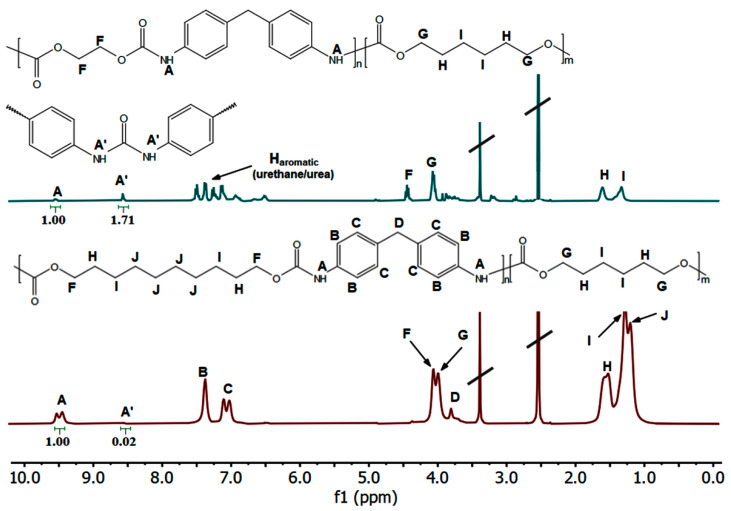
The comparison of the NIPCU_1_2 (**up**) and NIPCU_1_10 (**down**) structures based on ^1^H NMR spectroscopy.

The ^1^H NMR spectra of the NIPCUs were similar to the spectra of the corresponding BHACs. The only differences were the decrease in the integral intensity of the -CH_2_OH groups’ signals (4.80 ppm for the NIPCU_1_2 and 4.35 ppm for the remaining NIPCUs) corresponding to the increase in the final product’s molar mass, as well as the appearance of the -CH_2_OC(O)O signal from the NIPCU soft segments at about 4.00 ppm. However, in the case of NIPCU_1_4, NIPCU_1_5, and NIPCU_1_6, the signal from the -CH_2_OC(O)O groups overlapped with the signal from the -CH_2_OC(O)NH groups. Hence, their hard and soft segments were identified thanks to the other signals, e.g., at about 7.33–7.55 ppm (H_ar_ from the hard segments) and 1.28–1.33 ppm (methylene groups from the soft segments) (Figure 4 and Figures S11–S15).

Moreover, the ^1^H NMR spectra of the NIPCUs confirmed the length of the hydrocarbon chain in the terminal hydroxyalkyl carbamate groups, as well as the presence of urea groups (from BHAC), which had a high impact on the process of transurethane polycondensation, especially in terms of the formation of side products (urea bonds) via the backbiting reaction (Figure 2). The presence of short 2-hydroxyethyl- and 4-hydroxybutyl carbamate groups in the reaction mixture led to the formation of a high concentration of urea groups in the NIPCUs. The concentration of the urea bonds in the NIPCUs was calculated relative to the signal of the urethane groups (9.50 ppm) and equalled 46.03 mol% and 16.46 mol% for NIPCU_1_2 and NIPCU_1_4, respectively. These values were much higher compared to the corresponding BHACs.

In our previous studies, we proved that urea-free aliphatic and alicyclic NIPCUs could be obtained from bis(2-hydroxyalkyl carbamate)s devoid of urea groups. Moreover, the presence of OCD in the reaction mixture enabled the transformation of the urea groups into urethane ones, which was evidenced using model reactions [6,32]. However, here, the BHAC monomer with the 2-hydroxyethyl- or 4-hydroxybutyl carbamate groups and a high concentration of urea groups was used for the transurethane polycondensation. Hence, the presence of the urea groups at the initial stage of the reaction with OCD had a crucial impact on the generation of consecutive urea bonds. It was confirmed that the remaining NIPCUs (NIPCU_1_5, NIPCU_1_6, and NIPCU_1_10) obtained from the BHAC with a nonsignificant concentration of urea groups (1–2 mol%) contained similar concentrations of urea groups as the corresponding BHACs (1–2 mol%).

To calculate the concentrations of the urethane and carbonate groups from the hard and soft NIPCU segments and compare the calculated values with the theoretical ones, ^1^H NMR spectroscopy was used. The results are listed in Table 1 and the calculation methods are presented in Equations (S1) and (S2). In the case of NIPCU_1_2 and NIPCU_1_4, the calculated concentrations of the urethane and carbonate groups varied greatly compared to the theoretical ones due to the high content of urea groups that were formed that consumed the urethane groups. In the case of the remaining samples possessing low contents of urea moieties, the calculated values were consistent with the theoretical values.

Moreover, the molar mass of the NIPCUs was calculated based on ^1^H NMR spectroscopy, according to Equation (S3). The Gel Permeation Chromatography (GPC) method was not used, since the structure of the NIPCUs was far from that of the polystyrene standards. The calculated values of the NIPCUs’ molar masses are presented in Table 1. The lowest molar masses were obtained for NIPCU_1_2 and NIPCU_1_4, which contained high concentrations of urea groups, which increased the softening point of the NIPCUs and inhibited the progress of the reaction. These samples were also brittle, which macroscopically confirmed their low molar mass. In the case of the remaining NIPCUs, molar masses in the range of 20,000–28,000 g∙mol^−1^ were obtained.

The occurrence of the back-biting side reactions during transurethane polycondensation was confirmed by the investigation of the distillate structure using ^1^H NMR spectroscopy. However, only when the 2-hydroxyethyl or 4-hydroxybutyl carbamate groups were present in the system (NIPCU_1_2 or NIPCU_1_4), the ethylene carbonate (8.79 mol%) and MDA (0.79 mol%) or tetrahydrofuran (0.44 mol%) and MDA (0.44 mol%) were detected in the distillates, respectively. Moreover, the distillate of the NIPCU_1_2 or NIPCU_1_4 mainly contained 1,6-hexanediol (37.09 mol%) and 1,2-ethanediol (53.34 mol%), or 1,6-hexanediol (32.97 mol%) and 1,4-butanediol (66.16 mol%), respectively. In the case of the remaining samples, the distillates contained only 1,6-hexanediol and the appropriate α,ω-diol (1,5-pentanediol or 1,10-decanediol). This was evidence that the back-biting side reactions were preferred when the short hydroxyalkyl carbamate groups with less than five carbon atoms in the hydrocarbon chain were present in the system. The ^1^H NMR spectra of the distillates are shown in Figures S26–S30.

**Figure 5:**
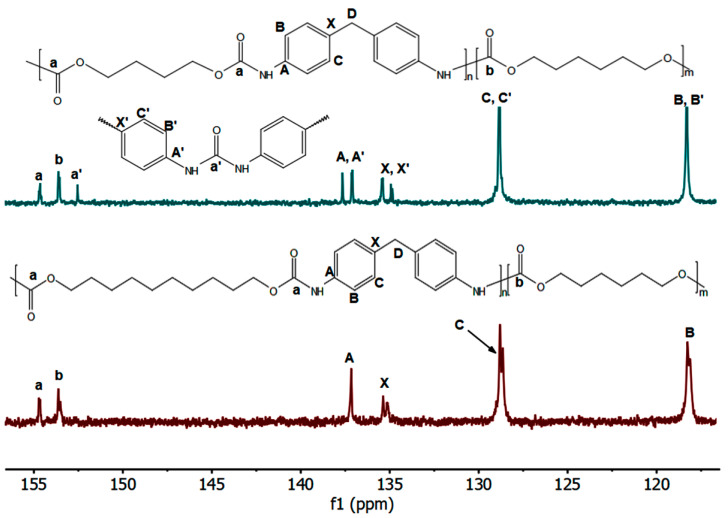
The comparison of the NIPCU_1_2 (**up**) and NIPCU_1_10 (**down**) structures based on ^13^C NMR spectroscopy.

The ^13^C NMR spectroscopy confirmed the presence of the urea bonds (152.56 ppm) in the case of NIPCU_1_2 and NIPCU_1_4 (e.g., Figure 5 and Figures S16–S20). The spectra of all NIPCUs showed the characteristic signals from urethane and carbonate’s carbonyl carbon atoms at about 154.72 ppm and 153.64 ppm, respectively. Moreover, the characteristic signals of -CH_2_OC(O)NH and -CH_2_OC(O)O were observed at about 63.92 ppm and 67.22 ppm, respectively, which confirmed the presence of urethane and carbonate bonds in the NIPCUs (Figures S16–S20).

The FT-IR spectra of NIPCU_1_2 and NIPCU_1_4 revealed an absorption band of hydrogen-bonded urea groups at about 1645 cm^−1^ [40]. The remaining samples were urea-free. The FT-IR spectra of all NIPCUs showed a broad and sharp absorption band at about 3320 cm^−1^ coming from the hydrogen-bonded NH group, consequently signifying the ordered arrangement of the NIPCU hard segments (Figures S21–S25). The strong and sharp absorption band at 1700–1730 cm^−1^ corresponded to the presence of hydrogen-bonded urethane groups and C=O stretching vibrations in the carbonate groups. The strong absorption bands at about 1520 cm^−1^ and 1220 cm^−1^ proved the existence of N-H bending and C-O-C asymmetric stretching vibrations.

Both the NMR and FT-IR spectroscopies proved that the obtained NIPCUs were free of allophanate and ether groups. The characteristic signals of allophanate (about 156 ppm at ^13^C NMR spectra) [41] and ether moieties (3.4–3.5 ppm at ^1^H NMR spectra) [42] were not observed. This proved that the crosslinking and decarboxylation side reactions were not preferred during transurethane polycondensation.

### 2.3. Thermal Properties of the Aliphatic–Aromatic NIPCUs

**Figure 6:**
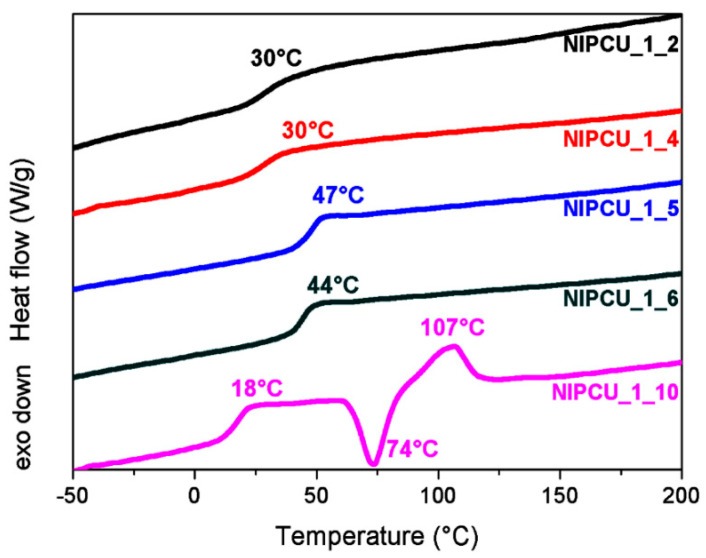
Thermal properties of the NIPCUs differing in their structures of hard segments.

The thermal properties of the aliphatic–aromatic NIPCUs differing in their structures of hard segments were evaluated using Differential Scanning Calorimetry (DSC). The DSC curves are shown in Figure 6 and the determined temperatures of the glass transition (*T_g_*), crystallization (*T_c_*), and melting (*T_m_*) are presented along with the corresponding values of enthalpies (Δ*H_c_* and Δ*H_c_*) in Table 2.

The DSC curves revealed the single glass transition and melting peaks, suggesting that the NIPCU hard (urethane) segments were at least partially miscible with the soft (carbonate) ones in the amorphous and crystalline phases. This was probably due to the formation of hydrogen bonds between them [17].

However, the aliphatic–aromatic NIPCUs obtained based on the BHAC with the shortest hydrocarbon chain in the hydroxyalkyl carbamate groups (2–6 carbon atoms) were fully amorphous. This resulted from the statistical structure of the obtained NIPCUs after the transurethane polycondensation of the monomeric BHAC with OCD. Only NIPCU_1_10 was semi-crystalline due to the presence of long decamethylene chains in the hard segments, which increased the order of the macromolecules and facilitated their crystallization.

Despite NIPCU_1_2 and NIPCU_1_4 containing the shortest hydrocarbon chains in the hard segments, they exhibited quite low values of *T_g_* due to the low molar mass. The stiffest macromolecules were obtained in the case of NIPCU_1_5 since it exhibited the highest value of *T_g_*. Shorter hydroxyalkyl carbamate moieties provided a higher density of the hydrogen bonds and hence increased the rigidity of the NIPCU chains. Moreover, when the number of carbon atoms in the hydrocarbon chains of the hard segments increased from 5 to 10, the *T_g_* decreased, whereas the crystalline phase content increased.

Our research team was the first to reveal the synthesis and characterization of aliphatic–aromatic NIPCUs [19]. The NIPCU (94 mol% of urethane groups) obtained from 4,4′-diphenylmethylene bis(5-hydroxypentyl carbamate) and amorphous oligo(hexamethylene-*co*-pentamethylene carbonate) diol was very stiff, amorphous, and exhibited a high *T_g_* value of 68 °C. In this work, the various amorphous or semi-crystalline aliphatic–aromatic NIPCUs containing 90 mol% of urethane segments of various structures were presented. The analogous sample synthesized based on crystalline oligo(hexamethylene carbonate) diol (NIPCU_1_5) was also amorphous but exhibited a lower *T_g_* value (47 °C) due to the presence of a lower concentration of urethane segments in the structure. More flexible NIPCU macromolecules were obtained when longer hydrocarbon chains (C6/C10-based) were present in the NIPCU hard segments (NIPCU_1_6 and NIPCU_1_10). All of the obtained NIPCUs were stiffer than typical aliphatic NIPCUs containing similar concentrations of urethane groups (96 mol%; *T_g_* of 9 °C) [19].

### 2.4. Mechanical Properties of the Aliphatic–Aromatic NIPCUs

**Figure 7:**
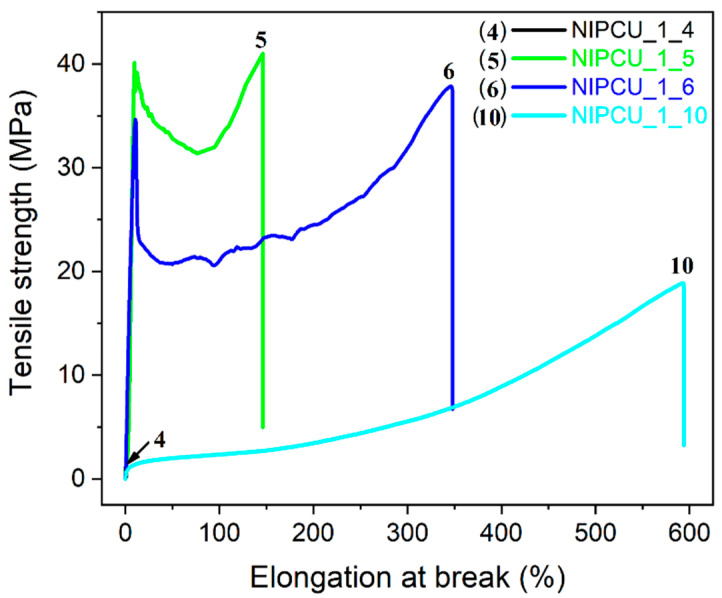
The strain–stress curves of NIPCUs differing in their structures of hard segments.

The mechanical properties of the aliphatic–aromatic NIPCUs differing in the length of the hydrocarbon chains (C2–C10-based) between the urethane groups in the hard segments were studied using tensile and hardness tests. The obtained strain–stress curves are presented in Figure 7, and the resulting values of the tensile strength, elongation at break, and hardness are given in Table 3.

The molar mass and length of the hydrocarbon chain separating the urethane groups in the hard segments greatly influenced the mechanical properties of the NIPCUs. Due to having the lowest number-average molar mass, NIPCU_1_2 was too brittle to be subjected to the stress tests. It also exhibited the lowest hardness. For the same reason, NIPCU_1_4 presented the lowest values of tensile strength, elongation at break, and hardness. In the case of the remaining samples of higher number-average molar masses, the increase in the number of carbon atoms (from 5 to 10) between the urethane groups in NIPCU hard segments resulted in the deterioration of the tensile strength and hardness, as well as the improvement of the flexibility. Increases in the NIPCU chains’ flexibilities were also observed based on the *T_g_* values. This was due to the weakened hydrogen bonds along the macrochains and worsened microphase separation. Therefore, the closer distribution of urethane groups promoted stronger hydrogen bonds between the hard and hard, as well as the hard and soft NIPCU segments, providing a high toughness, whereas the soft segments guaranteed typical elastomeric behavior.

In this work, aliphatic–aromatic NIPCUs of various mechanical properties were revealed. NIPCU_1_5 demonstrated a high tensile strength of 40 MPa and an elongation at break of 130%, whereas NIPCU_1_6 and NIPCU_1_10 containing longer alkyl chains in their hard segments displayed tensile strengths of 37 MPa and 18 MPa, and elongations at break of 300 and 550%, respectively. Thanks to both their toughness and flexibility, they are attractive alternatives for known NIPUs [6,19,22,37,38].

## 3. Materials and Methods

### 3.1. Materials

Dimethyl carbonate, MDA, 1,2-ethanediol (C2), 1,4-butanediol (C4), 1,5-pentanediol (C5), 1,6-hexanediol (C6), 1,10-decanediol (C10), acetonitrile, zinc acetate (dihydrate), and titanium(IV) butoxide were purchased from Merck (Poznan, Poland) and used as received. Oligo(hexamethylene carbonate) diol (ETERNACOLL UH200, OCD, number-average molar mass of 2000 g·mol^−1^) was purchased from UBE (Grao, Spain). OCD and α,ω-diols were dried under a vacuum prior to the NIPCU synthesis (1 h, 80 °C, 0.02 mbar).

### 3.2. Characterization Techniques

The ^1^H and ^13^C NMR spectra were recorded on a Varian VXR 400 MHz spectrometer using tetramethylsilane as an internal standard at 20 °C. The samples (15 mg for ^1^H NMR and 80 mg for ^13^C NMR) were dissolved in 1 mL of deuterated DMSO-d_6_.

FT-IR spectra were recorded on a Nicolet iS5 Mid Infrared FT-IR Spectrometer equipped with iD7 ATR Optical Base in the range of 4000–400 cm^−1^ at 32 scans and a resolution of 4 cm^−1^.

DSC measurements over the temperature range from −80 to 220 °C were performed on a TA Instrument DSC Q1000 calorimeter with heating and cooling rates of 10 °C∙min^−1^. The samples (10–25 mg) were first heated to 180 °C and then cooled to −80 °C to eliminate their thermal history. Then, they were heated to 220 °C at the same rate to study their thermal properties. Nitrogen was used as a purge gas (flow rate of 50 mL∙min^−1^).

The mechanical properties (tensile strength and elongation at break) of the samples were determined using an Instron 5566 testing machine with pneumatic grips and a 100 N head. Head speed was 50 mm∙min^−1^. The samples were paddle-shaped with a 12 mm length, 2 mm width, and about 1 mm thickness of the measuring segment. The tensile tests were performed according to the PN-EN ISO 527-2:2012 standard.

The hardness of the NIPCUs was determined by the Shore method according to the ISO R868:2005 standard. It was measured based on the resistance of the tested sample while driving the measuring needle into it. The measurements were made using a C.V. Instruments Ltd. Shore D Durometer hardness tester. The penetration time of the indenter was 5 s, whereas the resolution of the device was 1 on the Shore D scale. The obtained results were averaged based on 10 measurements at different locations of the sample.

### 3.3. Syntheses

#### 3.3.1. Synthesis of Arylene BMC

The 4,4′-diphenylmethylene BMC (1) was obtained according to the modified procedures reported by Reixach et al. [30] The MDA (0.06 mol), dimethyl carbonate (0.90 mol), and zinc acetate (0.0006 mol, 1 mol%) were placed into a 200 mL stainless steel autoclave and mixed using a magnetic stirrer (800 rpm). Then, the atmosphere was purged by nitrogen flow and the reaction was carried out at 180 °C for 2 h. Afterward, the autoclave was cooled to about 20 °C. Finally, the white solid was filtered off and dried under a vacuum. The product was crystallized from hot acetonitrile. Yield = 94%, mp = 182–183 °C.

For ^1^H NMR (400 MHz, DMSO-d_6_), δ (ppm) = 3.64 (s, OCH_3_, 6H,), 3.79 (s, CH_2_, 2H), 7.09–7.11 (d, J = 8.0 Hz, H_Ar_, 4H,), 7.34–7.36 (d, J = 8.0 Hz, H_Ar_, 4H,), 9.54 (bs, NH, 2H,). For ^13^C NMR (100 MHz, DMSO-d_6_), δ (ppm) = 40.15 (CH_2_), 51.58 (OCH_3_), 118.44 (C_Ar_), 128.94 (C_Ar_), 135.59 (C_Ar_), 137.17 (C_Ar_), 154.47 (OC(O)O). FT-IR (ATR): 3326, 2951, 1707, 1602, 1532, 1438, 1413, 1322, 1235, 1072, 825, 772, 642, 514 cm^−1^.

#### 3.3.2. Transurethanization of the Arylene BMC with α,ω-diol (C2, C4, C5, C6, or C10)

The transurethanization toward aliphatic–aromatic BHACs (1_2; 1_4; 1_5; 1_6; and 1_10) was carried out according to the modified procedure reported by our research group [19]. The reactants (BMC and C2, C4, C5, C6, or C10 diol) and catalyst (zinc acetate, 1.0 mol% referring to the BMC) were placed into the four-necked reactor equipped with a nitrogen inlet, a fractionating column with a Liebig condenser, thermometer, and a mechanical stirrer. The mixture was stirred at 180 °C until the methoxycarbonyl group’s signal (3.6 ppm) disappeared in the ^1^H NMR spectra (about 3 h). The molar ratio of the arylene BMC to the α,ω-diol was 1/3. During this reaction, low-molar-mass products were removed from the system thanks to the nitrogen flow. The final BHACs were used as obtained, without purification for the transurethane polycondensation process. An excess of appropriate α,ω-diol was distilled off during the initial stage of the transurethane polycondensation. The amounts of chemicals used for the preparation of the BHACs are presented in Table S1. The ^1^H NMR spectra of the BHACs (1_2, 1_4, 1_5, 1_6, and 1_10) and the corresponding distillates are presented in Figures S1–S10.

4,4′-diphenylmethylene bis(10-hydroxydecyl carbamate) (product 1_10): ^1^H NMR (400 MHz, DMSO-d_6_): δ (ppm) = 1.26 (m, CH_2_, 13.5 H), 1.39 (m, CH_2_, 2.3 H), 1.56 (m, CH_2_, 2.3 H), 3.77 (s, CH_2_, 1.0 H), 4.04 (t, J = 6.6 MHz, CH_2_OC(O)NH, 2.3 H), 4.32 (t, J_1_ = 5.2 MHz, CH_2_OH, 2.3 H), 4.84 (bs, OH, 0.2 H), 7.06–7.08 (d, J = 8.4 MHz, H_Ar_, 2.3 H), 7.33–7.35 (d, J = 8.0 MHz, H_Ar_, 2.3 H), 8.49 (s, NHC(O)NH, 0.02 H), 9.48 (bs, NHC(O)O, 1.0 H).

#### 3.3.3. Synthesis of the Aliphatic–Aromatic NIPCUs

The transurethane polycondensation reactions of the previously obtained aliphatic–aromatic BHAC (90 mol%) with OCD (10 mol% of crystalline oligo(hexamethylene carbonate) diol) were carried out in a three-necked reactor equipped with a Liebig condenser, a thermometer, and a mechanical stirrer. The syntheses were carried out at 180 °C under reduced pressure (ca. 0.02 mbar) in the presence of titanium(IV) butoxide (1 mol% referred to as OCD) as a catalyst until the further diffusion of low-molar-mass diols from the system was not observed. Then, the NIPCUs were poured off on a Teflon^®^ plate and cooled to room temperature (about 20 °C). Finally, the samples were hot-pressed at 180 °C using a hydraulic press to produce NIPCU thin films for the mechanical properties evaluation. The amounts of BHAC, OCD, and catalyst used for the preparation of the NIPCUs are presented in Table S2. The structure of the NIPCUs was studied using ^1^H and ^13^C NMR as well as FT-IR spectroscopies. The obtained results are listed in Figures S11–S25 and the ^1^H NMR spectra of the NIPCUs’ distillates are presented in Figures S26–S30. 

NIPCU_1_10: ^1^H NMR (400 MHz, DMSO-d_6_): δ (ppm) = 1.16 (CH_2_, 437.6 H), 1.24 (CH_2_, 432.8 H), 1.49–1.54 (CH_2_, 428.1 H), 3.77 (CH_2_, 37.3 H), 3.95 (CH_2_OC(O)NH, 204.9 H), 4.02 (CH_2_OC(O)O, 164.4 H), 4.33 (CH_2_OH, 4.0 H), 6.99–7.07 (H_Ar_, 163.7 H), 7.33 (H_Ar_, 160.4 H), 8.50 (NHC(O)NH, 1.6 H), 9.42–9.50 (NHC(O)O, 76.8 H). ^13^C NMR (100 MHz, DMSO-d_6_): δ (ppm) = 24.80–28.89 (CH_2_), 40.15 (CH_2_), 64.03 (CH_2_OC(O)NH), 67.31 (CH_2_OC(O)O), 118.26 (C_Ar_), 128.64 (C_Ar_), 135.38 (C_Ar_), 137.16 (C_Ar_), 153.63 (OC(O)O), 154.70 (OC(O)NH). FT-IR (ATR): 3334, 2926, 2854, 1702, 1599, 1538, 1471, 1410, 1255, 1222, 1063 cm^−1^.

## 4. Conclusions

In this study, aliphatic–aromatic NIPCUs differing in their structures of hard segments were obtained in three main steps: the methoxycarbonylation of MDA, the transurethanization of 4,4′-diphenylmethylene BMC with α,ω-diol (C2–C10), and the transurethane polycondensation of 4,4′-diphenylmethylene BHAC with crystalline oligo(hexamethylene carbonate) diol.

The influence of the α,ω-diol alkyl chain length (2, 4, 5, 6, or 10 carbon atoms) on the process of transurethanization with 4,4′-diphenylmethylene BMC was studied thanks to the analysis of the BHAC products and distillate structures using ^1^H NMR spectroscopy. The strong susceptibility of hydroxyethyl and hydroxybutyl carbamate moieties (1_2 and 1_4) to the intramolecular side reactions (back-biting) was observed due to the formation of thermodynamically stable cyclic products (ethylene carbonate or tetrahydrofuran, respectively). The consequences of these reactions were the presence of urea bonds in the BHACs and the detection of the proper cyclic products in the final products and distillates. When a longer alkyl chain (hydroxypentyl, hydroxyhexyl, or hydroxydecyl carbamate) was introduced into the BHAC structure, it was not prone to the backbiting side reaction due to the absence of the possibility of the formation of thermodynamically stable cyclic products.

The obtained bifunctional hydroxyl-terminated molecules differing in their alkyl chain lengths (1_2, 1_4, 1_5, 1_6, or 1_10) were used as the NIPCU hard segment precursors during non-solvent transurethane polycondensation with the 10 mol% of oligo(hexamethylene carbonate) diol (NIPCU soft segment precursor) yielding diverse aliphatic–aromatic NIPCU with C2–C10-based hard segments. The susceptibility of the hydroxyalkyl carbamate moieties to the back-biting side reaction was the same as in the case of the reaction with low-molar-mass α,ω-diol. The back-biting side reactions were preferred when the short hydroxyalkyl carbamate groups, having less than five carbon atoms in the hydrocarbon chain, were present in the system. Importantly, the presence of a high concentration of urea groups in the BHAC promoted the further formation of urea groups during the transurethane polycondensation with OCD.

Both ^1^H and ^13^C NMR, as well as FT-IR spectroscopies, proved that the obtained NIPCUs were free of allophanate and ether groups. Moreover, these techniques explicitly confirmed the presence of carbonate and urethane (and urea for some of the samples) bonds.

The thermal and mechanical properties of the NIPCUs were studied using DSC measurements as well as tensile and hardness tests. An increase in the hydrocarbon chain length (from 5 to 10 carbon atoms) between the urethane groups in the NIPCU hard segments resulted in a decrease in the macromolecules’ rigidity. Then, an increase in the elongation at break and decreases in the *T_g_*, tensile strength and hardness were observed. Moreover, an enhancement of the crystalline phase content was detected. Only NIPCU_1_10 was semi-crystalline, whereas the remaining NIPCUs were amorphous.

The obtained NIPCUs exhibited excellent mechanical properties (e.g., a tensile strength of 40 MPa and an elongation at break of 130%) that were similar to those of conventional isocyanate-based PCUs. Depending on the structures of the hard segments, they can be applied as rigid and/or flexible polymer materials. Moreover, due to their thermoplastic properties, they can be easily processed using typical methods for thermoplastics.

## Figures and Tables

**Table 1:** Theoretical and calculated values of the concentrations of the urethane and carbonate groups, as well as the number-average molar masses of the NIPCUs.

	Urethane Groups		Carbonate Groups		${\bar{M}}_{n}\;*$
	Theor.	Calc.	Theor.	Calc.	Calc.
	/mol%	/mol%	/mol%	/mol%	g·mol^−1^
NIPCU_1_2	*57.93*	26.51	*42.07*	73.49	6000
NIPCU_1_4	*57.93*	47.38	*42.07*	52.62	15,000
NIPCU_1_5	*57.93*	54.47	*42.07*	45.53	20,000
NIPCU_1_6	*57.93*	55.06	*42.07*	44.94	28,000
NIPCU_1_10	*57.93*	55.49	*42.07*	44.51	24,000

*—The number-average molar mass calculated using ^1^H NMR spectroscopy.

**Table 2:** Thermal properties of the obtained NIPCUs.

Sample	*T_g_* /°C	*T_c_* /°C	Δ*H_c_* /J∙g^−1^	*T_m_* /°C	Δ*H_m_* /J∙g^−1^
NIPCU_1_2	30	-	-	-	-
NIPCU_1_4	30	-	-	-	-
NIPCU_1_5	47	-	-	-	-
NIPCU_1_6	44	-	-	-	-
NIPCU_1_10	18	74	9	107	11

**Table 3:** The mechanical properties of the obtained **NIPCUs**.

NIPCU	Tensile Strength /MPa	Elongation at Break /%	Hardness (Shore D)
NIPCU_1_2	- ^a^	- ^a^	44 ± 4
NIPCU_1_4	3.1 ± 0.5	0.8 ± 0.3	57 ± 2
NIPCU_1_5	40.2 ± 1.3	130 ± 60	75 ± 2
NIPCU_1_6	37.4 ± 1.1	300 ± 40	72 ± 3
NIPCU_1_10	18.1 ± 0.7	550 ± 30	54 ± 4

^a^—the sample was too brittle to take the measurements.

## Data Availability

Not applicable.

## Supplementary Materials

The following supporting information can be downloaded at: https://www.mdpi.com/article/10.3390/ijms231910999/s1.
