# Supplementary material for: Non-Isocyanate Aliphatic–Aromatic Poly(carbonate-urethane)s—An Insight into Transurethanization Reactions and Structure–Property Relationships
Source: Int J Mol Sci. 2022 Sep 20;23(19):10999. doi: 10.3390/ijms231910999 (PMC9570502; doi:10.3390/ijms231910999)
Supplement: Supplementary file 1 [file ijms-23-10999-s001.zip › ijms-1906886-supplementary.pdf]

Supplementary Materials

Non-Isocyanate Aliphatic–Aromatic Poly(carbonate-urethane)s—An Insight into Transurethanization Reactions and Structure–Property Relationships

Faculty of Chemistry, Warsaw University of Technology, Noakowskiego 3,

00-664 Warsaw, Poland

Correspondence: dominik.wolosz.dokt@pw.edu.pl

1. Transurethanization of the arylene bis(methyl carbamate) with α,ω-diol (C2, C4, C5, C6, or C10)

The amounts of chemicals used for the preparation of the aliphatic-aromatic bis(hydroxyalkyl carbamate) diols (BHACs) were listed in Table S1.

Table S1. The amounts of chemicals used for the preparation of BHACs.

Symbol of BHAC	BMC[a]		α,ω-diol			zinc acetate
	n /mol	m /g	name	n /mol	m /g	m /g
**1_2**	0.050	**15.70**	1,2-ethanediol	0.150	**9.30**	**0.093**
**1_4**	0.048	**15.20**	1,4-butanediol	0.145	**13.07**	**0.090**
**1_5**	0.046	**14.35**	1,5-pentanediol	0.137	**14.26**	**0.085**
**1_6**	0.046	**14.44**	1,6-hexanediol	0.138	**16.29**	**0.085**
**1_10**	0.043	**13.50**	1,10-decanediol	0.129	**22.45**	**0.080**

[a] – 4.4′-diphenylmethylene bis(methyl carbamate).

The structure of the obtained BHACs was studied by means of ^{1}H NMR spectroscopy. The obtained spectra are presented in Figures S1–S5.

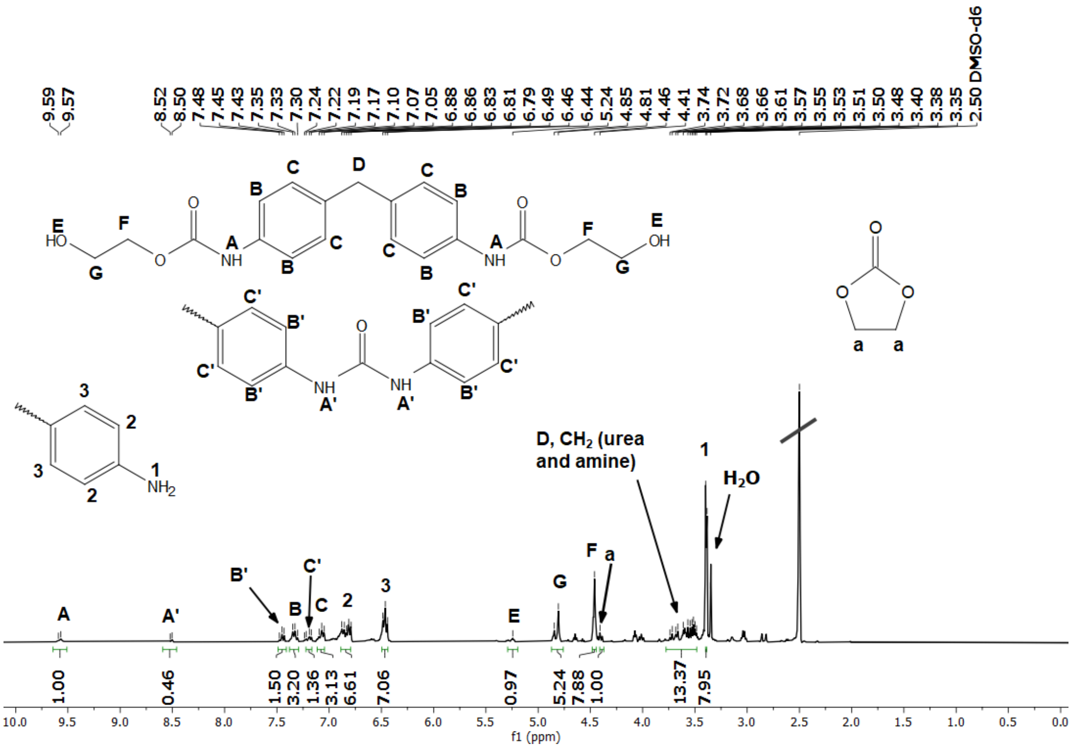

Figure S1. ^{1}H NMR spectrum of 1_2 product in DMSO-d$_6$.

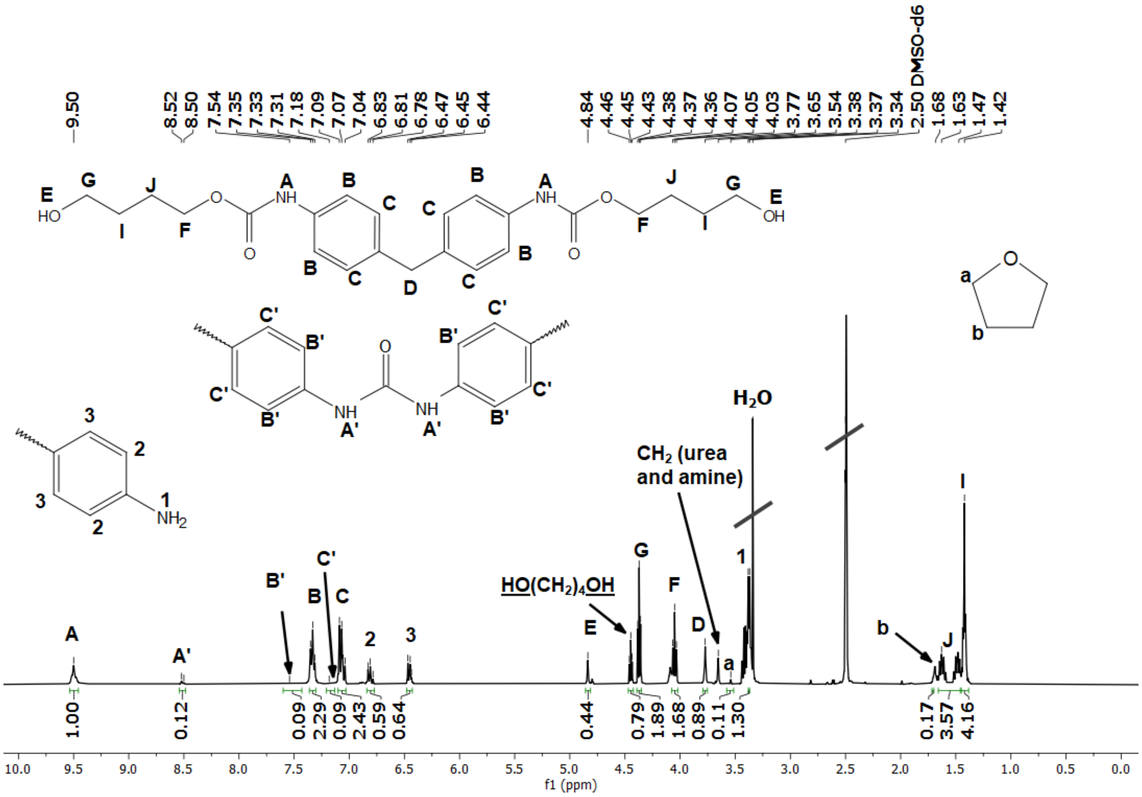

Figure S2. ^1H NMR spectrum of 1_4 product in DMSO-d_6.

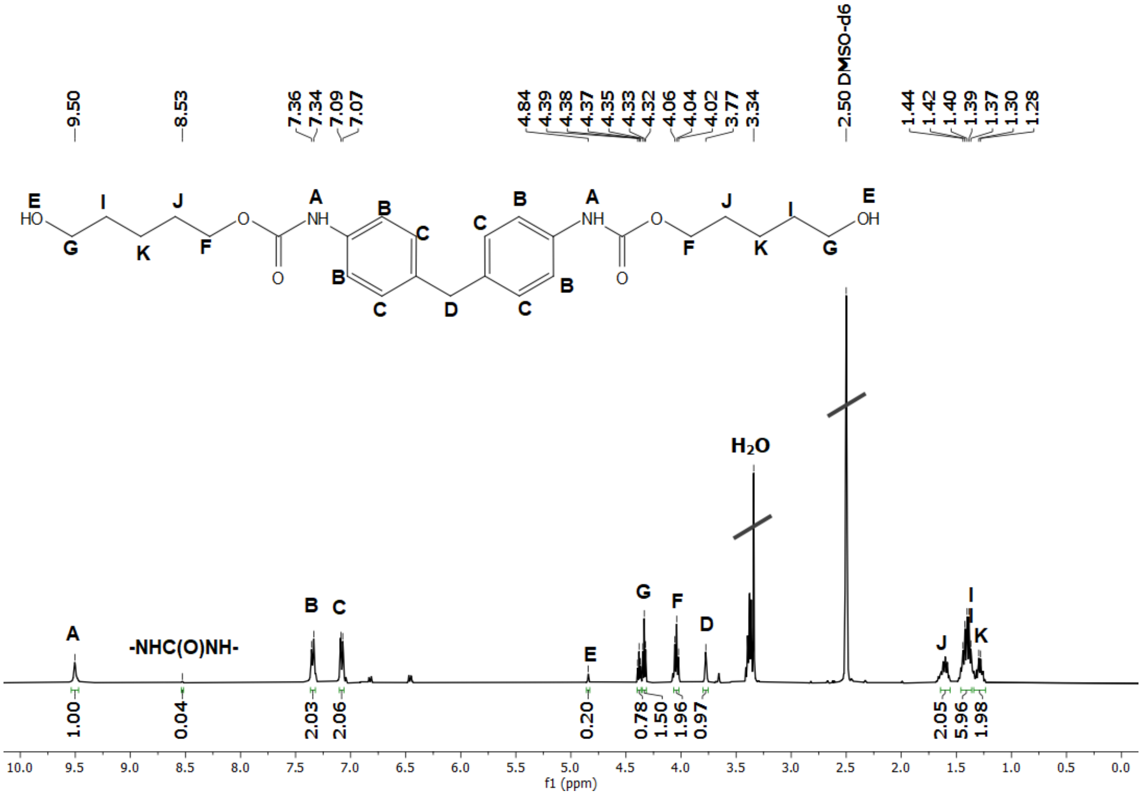

Figure S3. ^1H NMR spectrum of 1_5 product in DMSO-d_6.

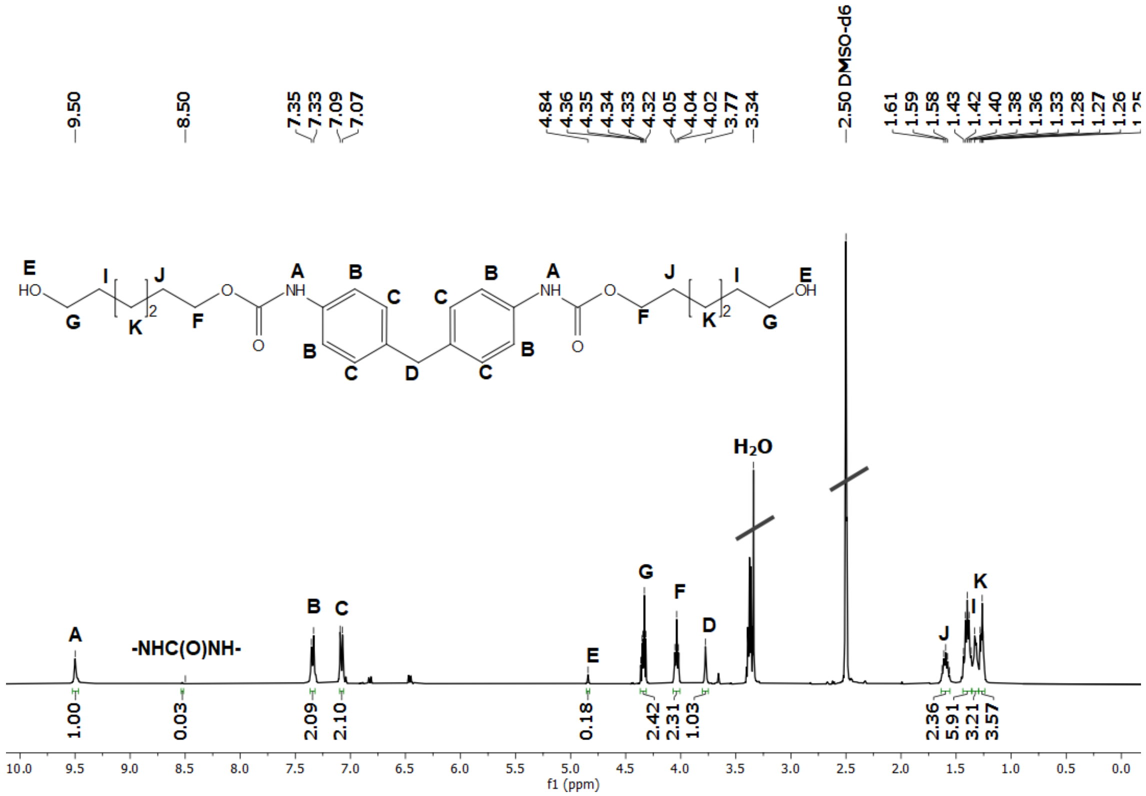

Figure S4. ^{1}H NMR spectrum of 1_6 product in DMSO-d6.

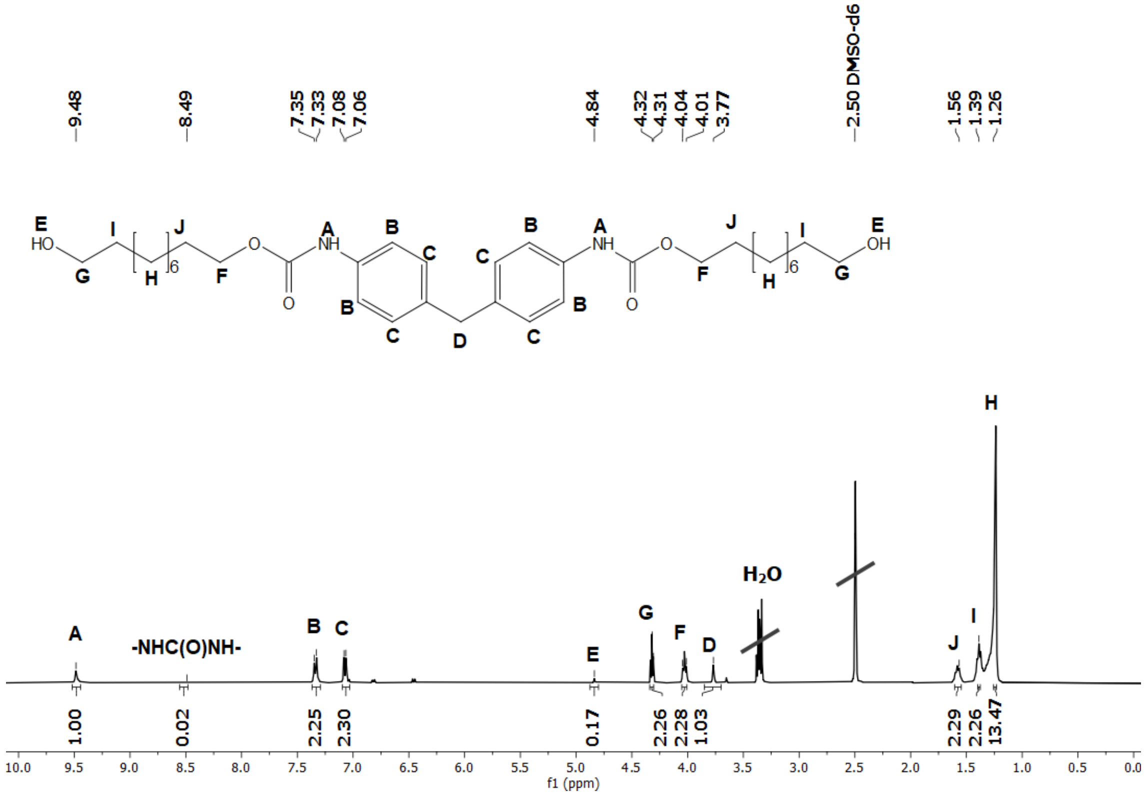

Figure S5. ^{1}H NMR spectrum of 1_10 product in DMSO-d6.

The structure of the distillates obtained after the transurethanization was studied by means of ^{1}H NMR spectroscopy. The spectra are shown in Figures S6–S10.

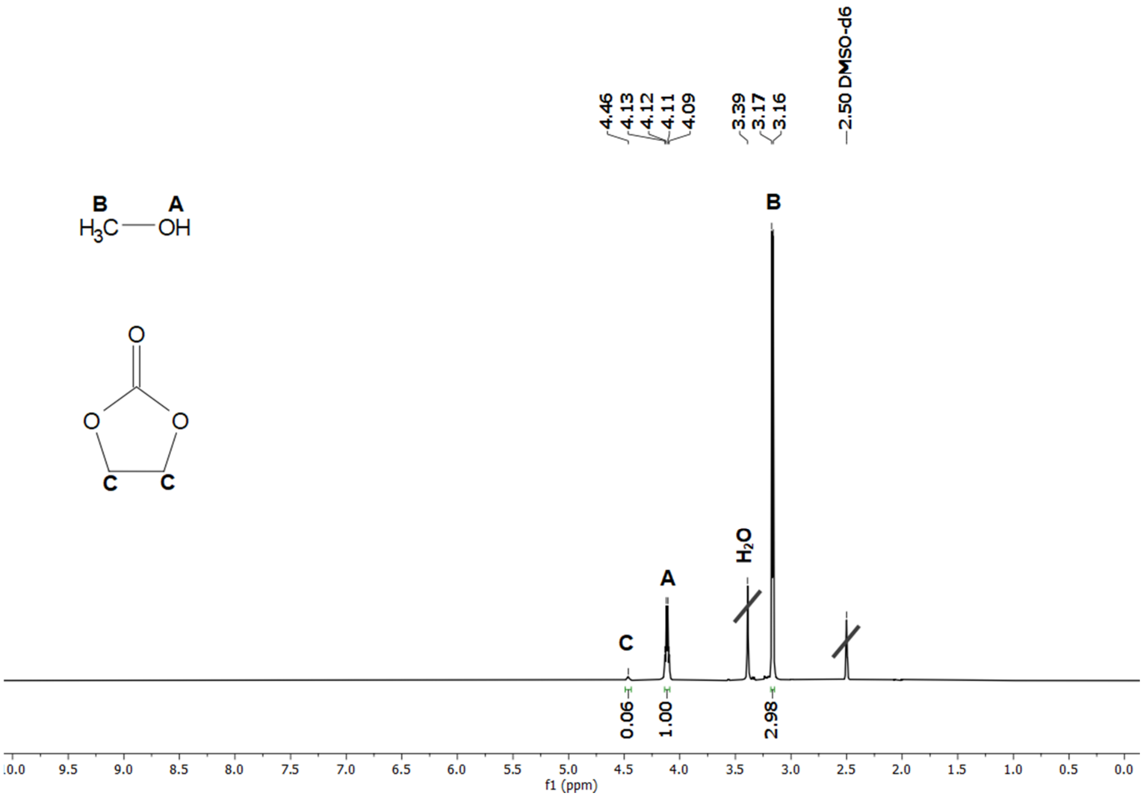

Figure S6. ^{1}H NMR spectrum of the 1_2′s distillate.

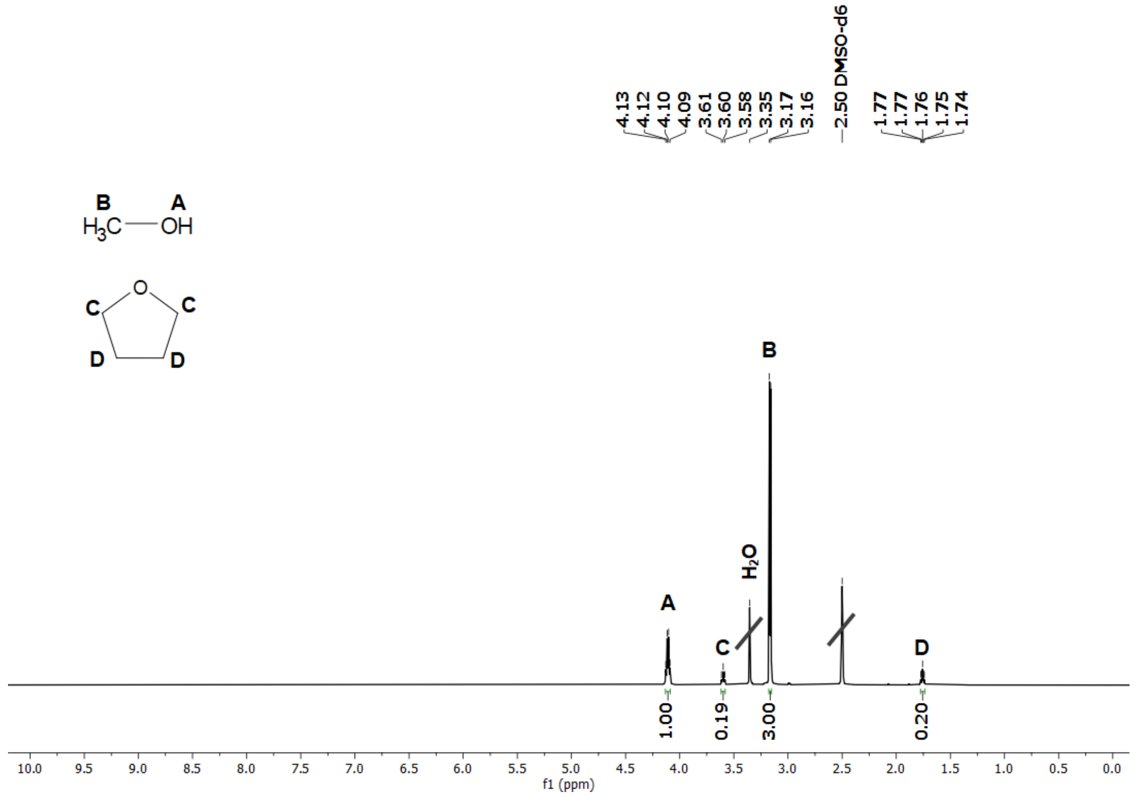

Figure S7. ^{1}H NMR spectrum of the 1_4′s distillate.

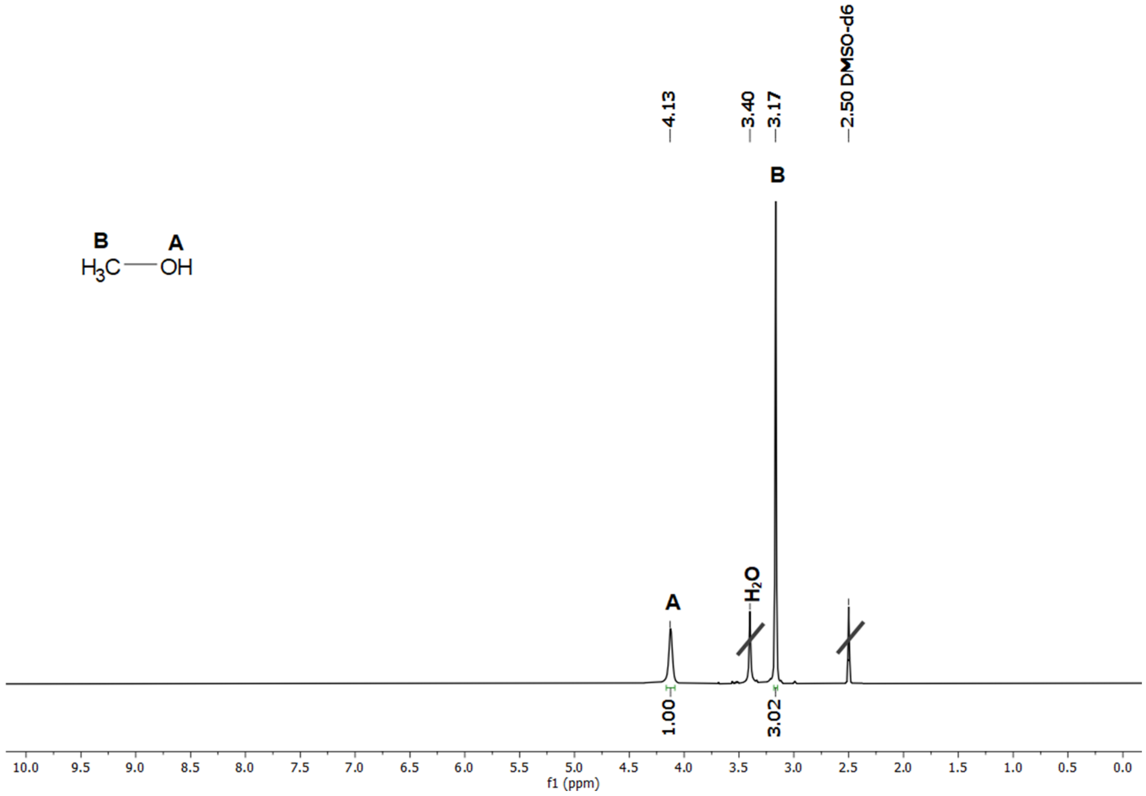

Figure S8. ^{1}H NMR spectrum of the 1_5′s distillate.

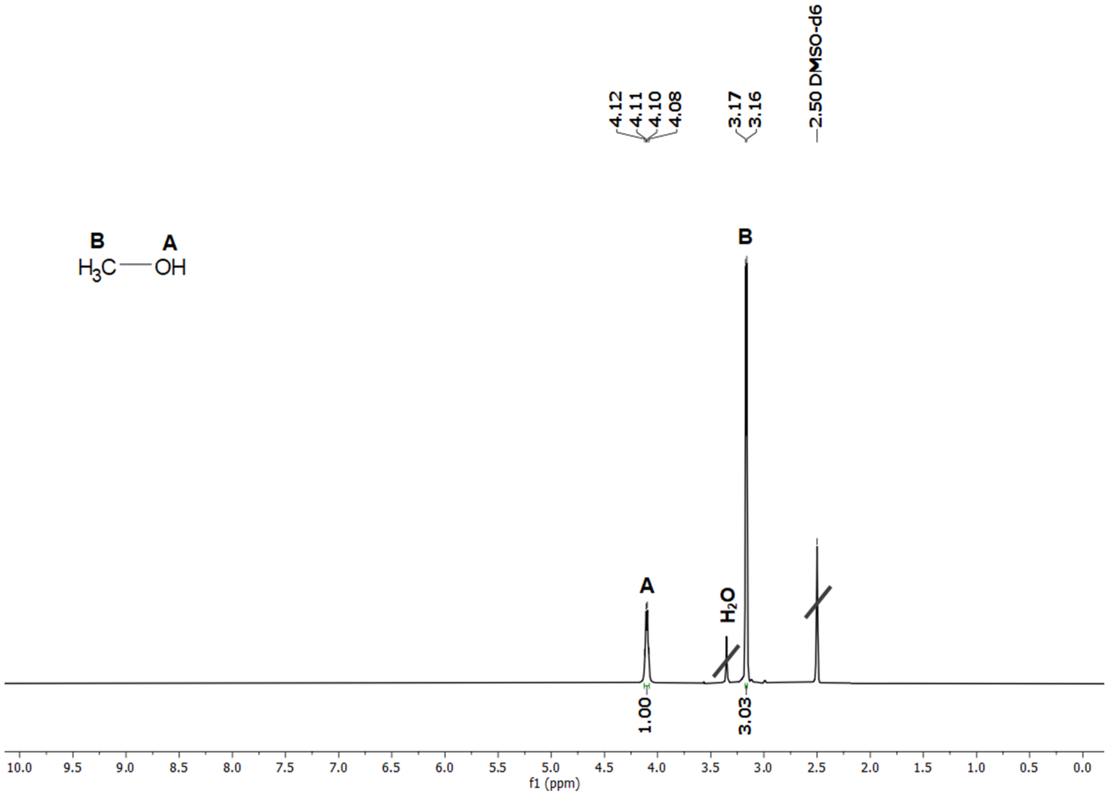

Figure S9. ^{1}H NMR spectrum of the 1_6′s distillate.

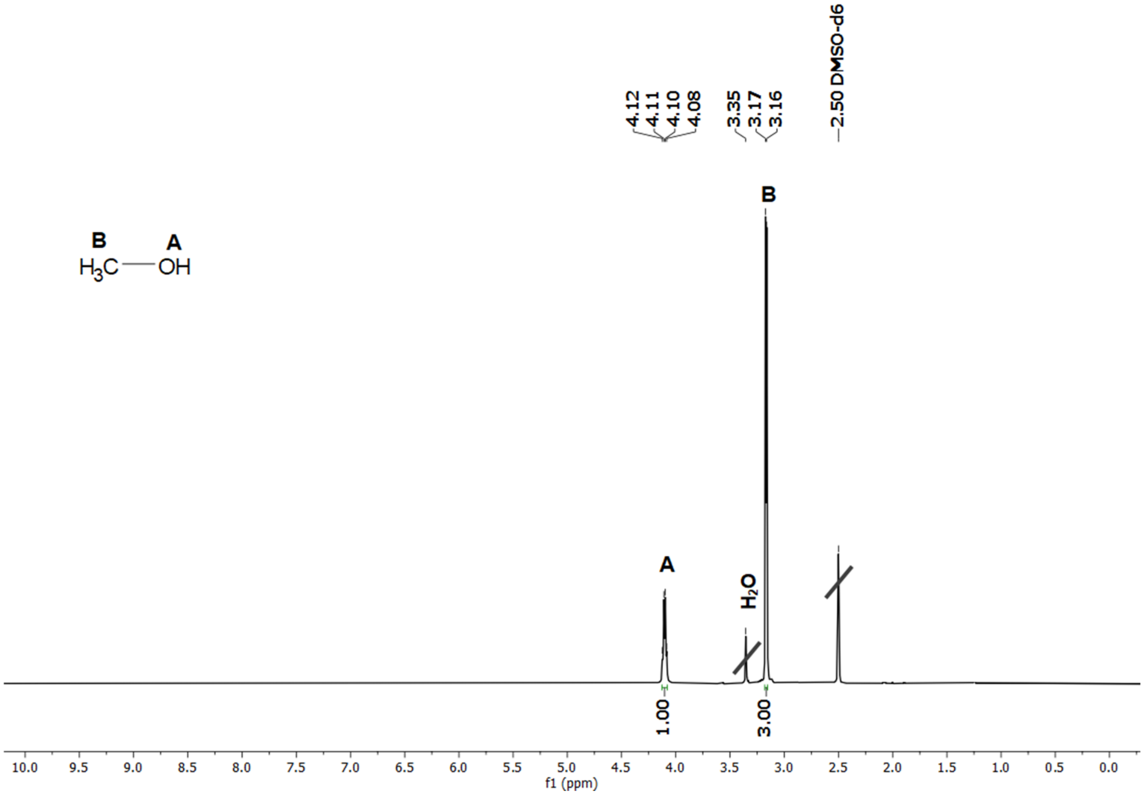

Figure S10. ^{1}H NMR spectrum of the 1_10′s distillate.

2. Synthesis of the non-isocyanate aliphatic-aromatic poly(carbonate-urethane)s (NIPCUs)

The amounts of BHAC, oligocarbonate diol (OCD), and the catalyst used for the preparation of NIPCUs were presented in the supplementary materials (Table S2).

Table S2. The amounts of the reagents and catalyst used for the preparation of NIPCUs with the 90 mol% of BHAC in feed.

NIPCU	BHAC		OCD		titanium(IV) butoxide
	n /mol	m* /g	n /mol	m /g	m /g
**NIPCU_1_2**	0.050	**18.699**	0.006	**11.099**	**0.019**
**NIPCU_1_4**	0.048	**20.816**	0.005	**10.746**	**0.018**
**NIPCU_1_5**	0.046	**20.933**	0.005	**10.145**	**0.017**
**NIPCU_1_6**	0.046	**22.353**	0.005	**10.208**	**0.017**
**NIPCU_1_10**	0.043	**25.716**	0.005	**9.544**	**0.016**

*—theoretical amount of BHAC after the removing of molar excess of the proper α,ω-diol.

The structure of the NIPCUs was studied by means of ^{1}H and ^{13}C NMR, as well as FT-IR spectroscopies. Obtained results were presented in Figures S11–S25 while the ^{1}H NMR spectra of the NIPCUs' distillates were shown in Figures S26–S30 (supplementary materials).

2.1. ^{1}H NMR spectra of NIPCUs

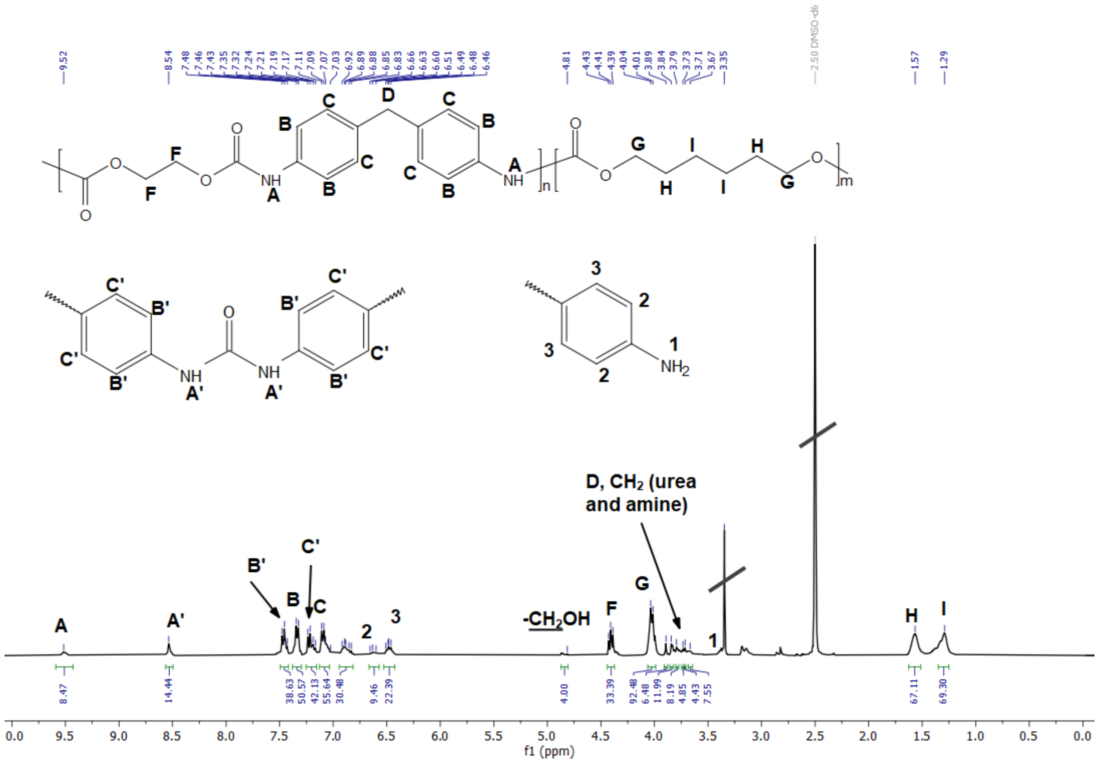

Figure S11. ^{1}H NMR spectra of NIPCU_1_2.

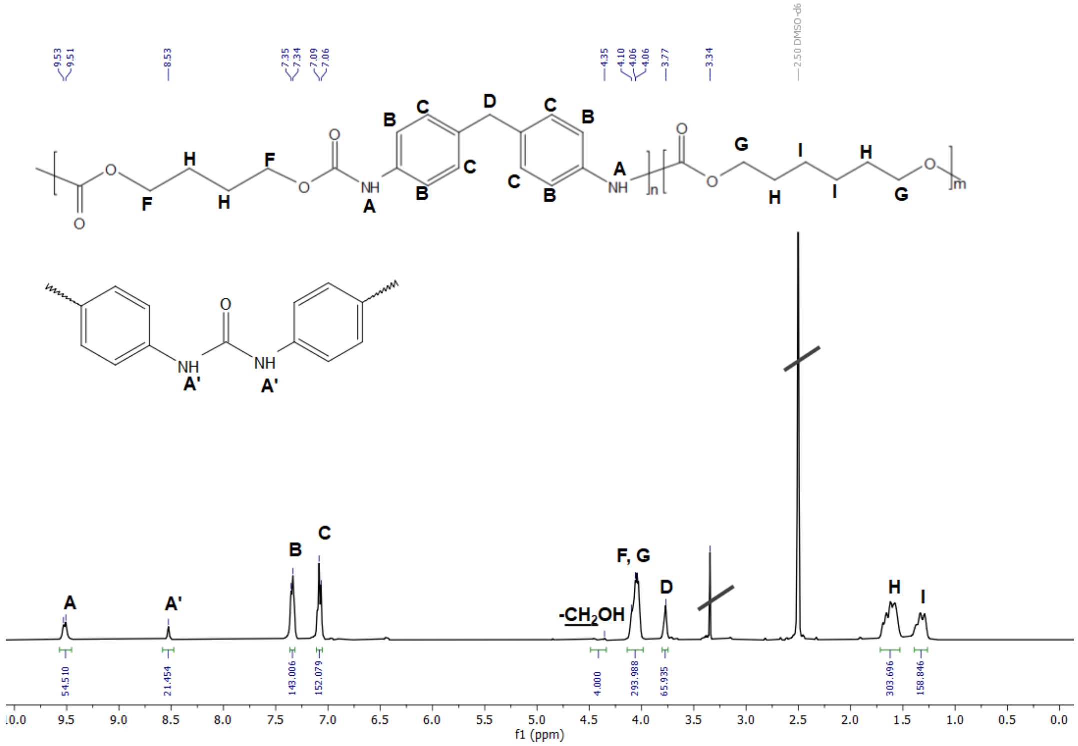

Figure S12. ^{1}H NMR spectra of NIPCU_1_4.

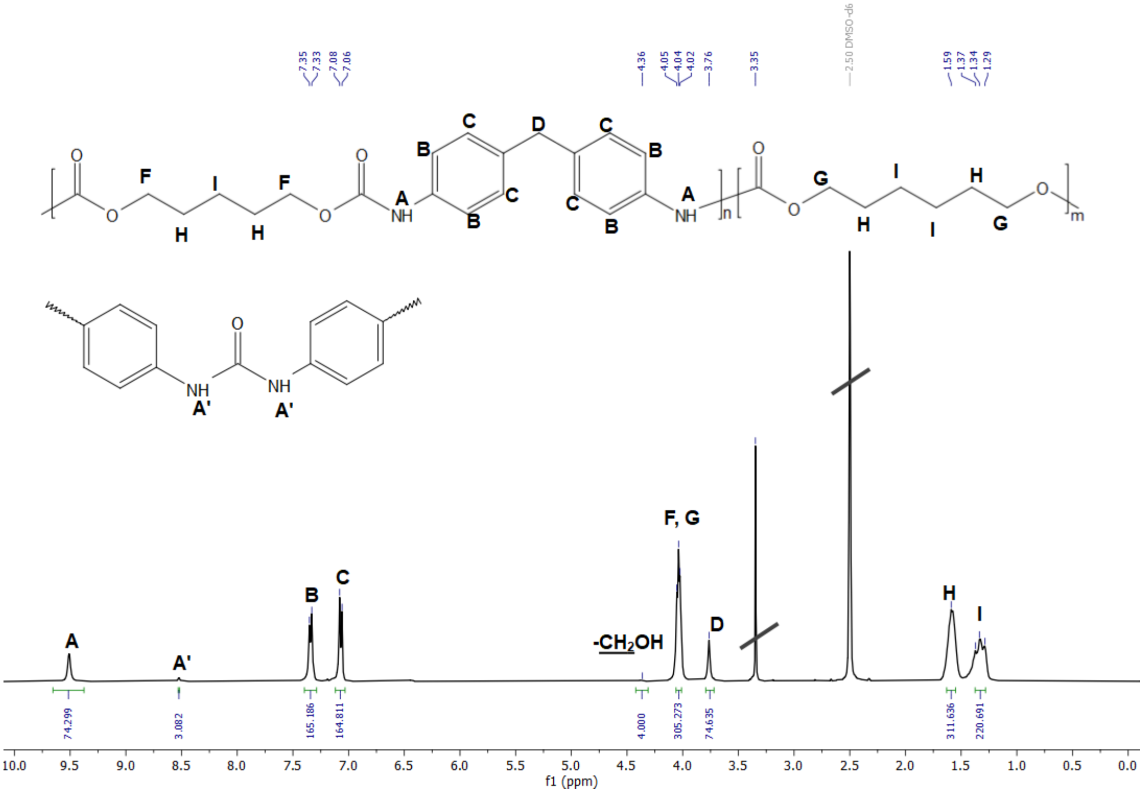

Figure S13. ^{1}H NMR spectra of NIPCU_1_5.

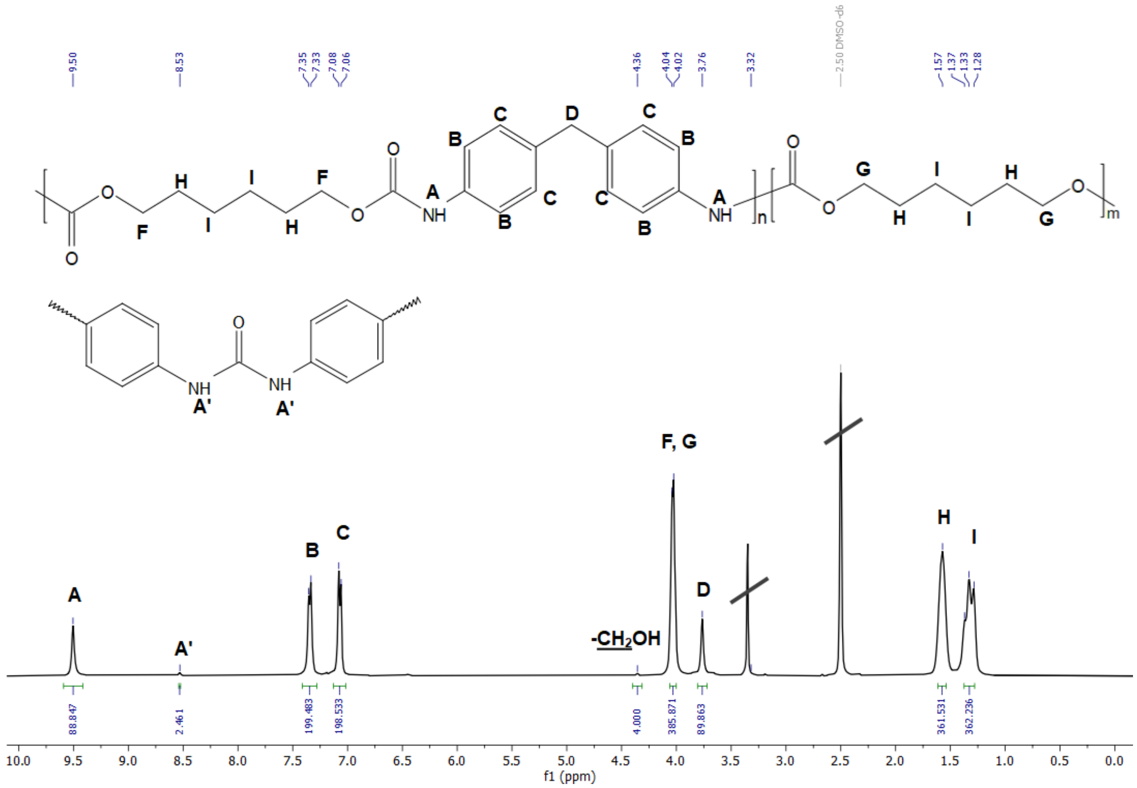

Figure S14. ^{1}H NMR spectra of NIPCU_1_6.

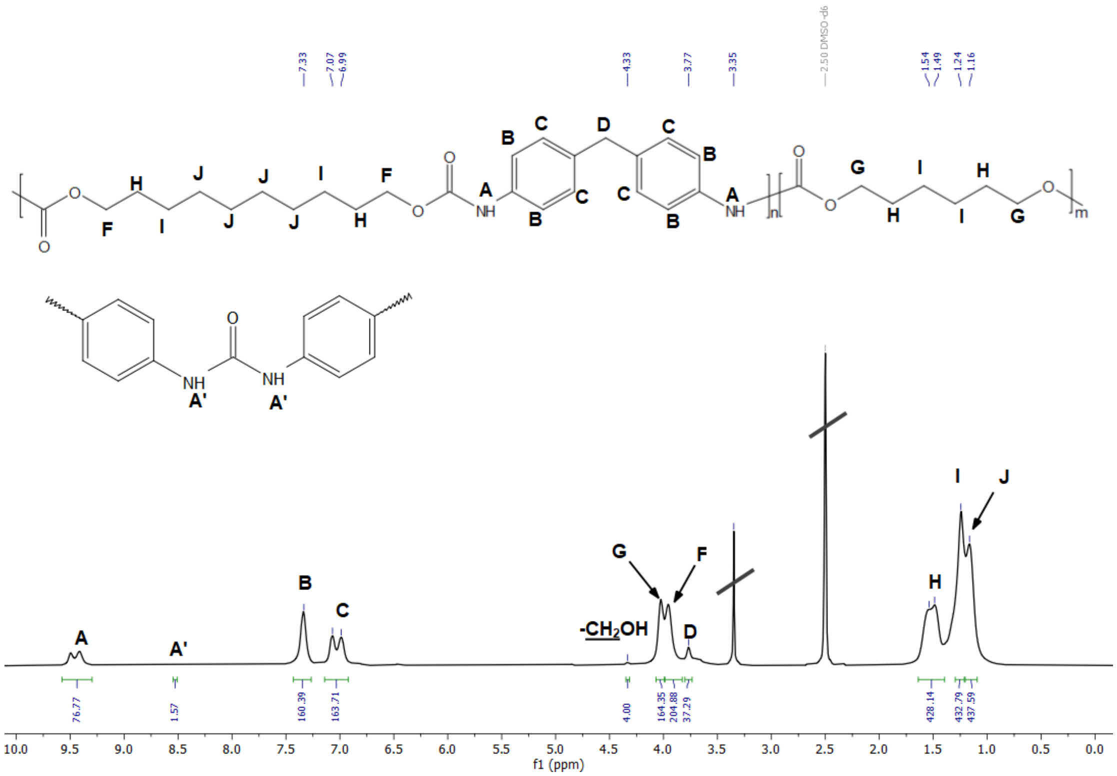

Figure S15. ^{1}H NMR spectra of NIPCU_1_10.

2.2. ^{13}C NMR spectra of NIPCUs

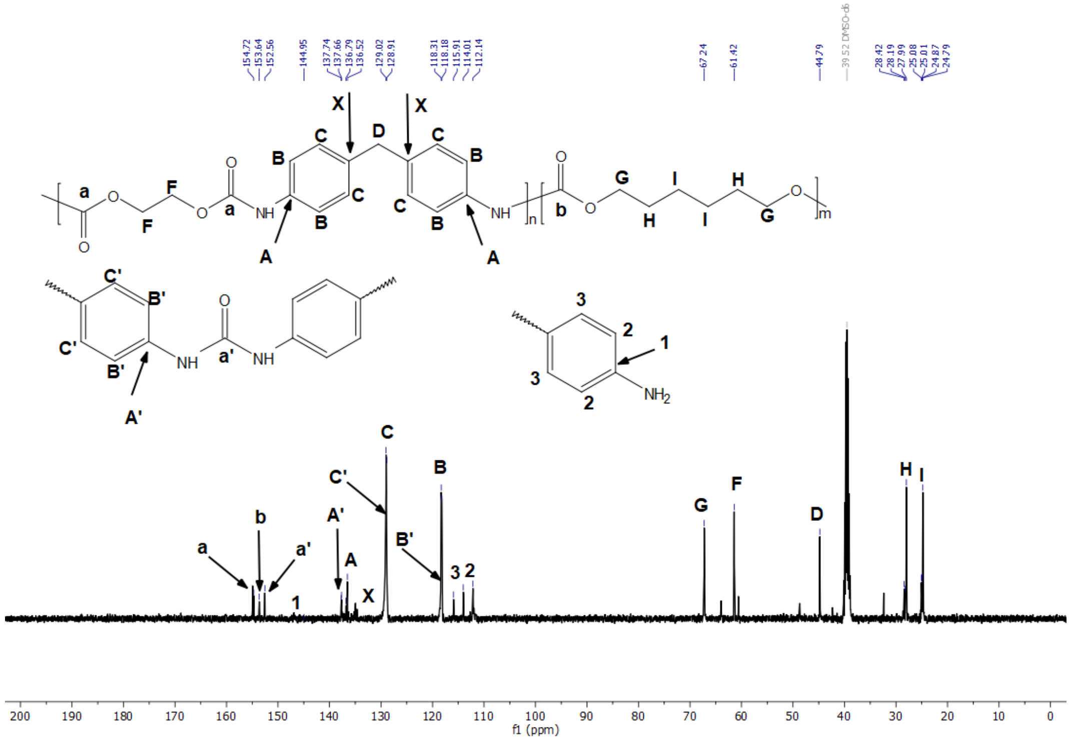

Figure S16. ^{13}C NMR spectra of NIPCU_1_2.

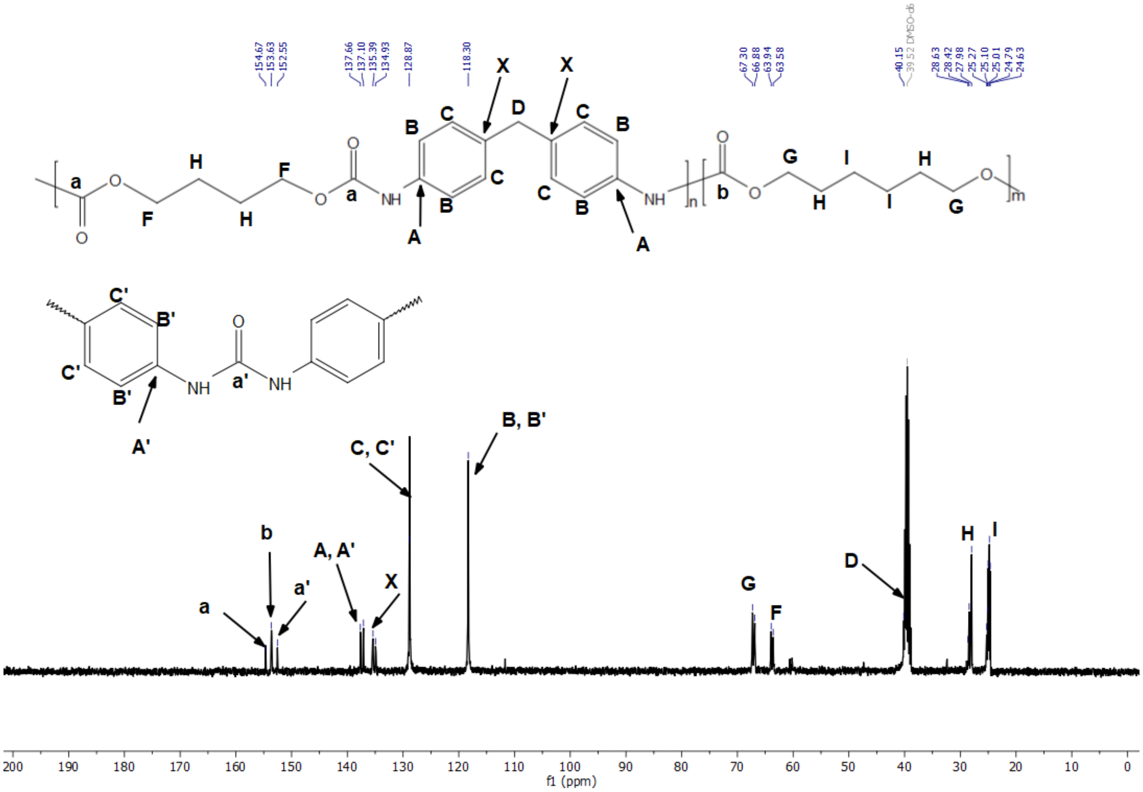

Figure S17. ^{13}C NMR spectra of NIPCU_1_4.

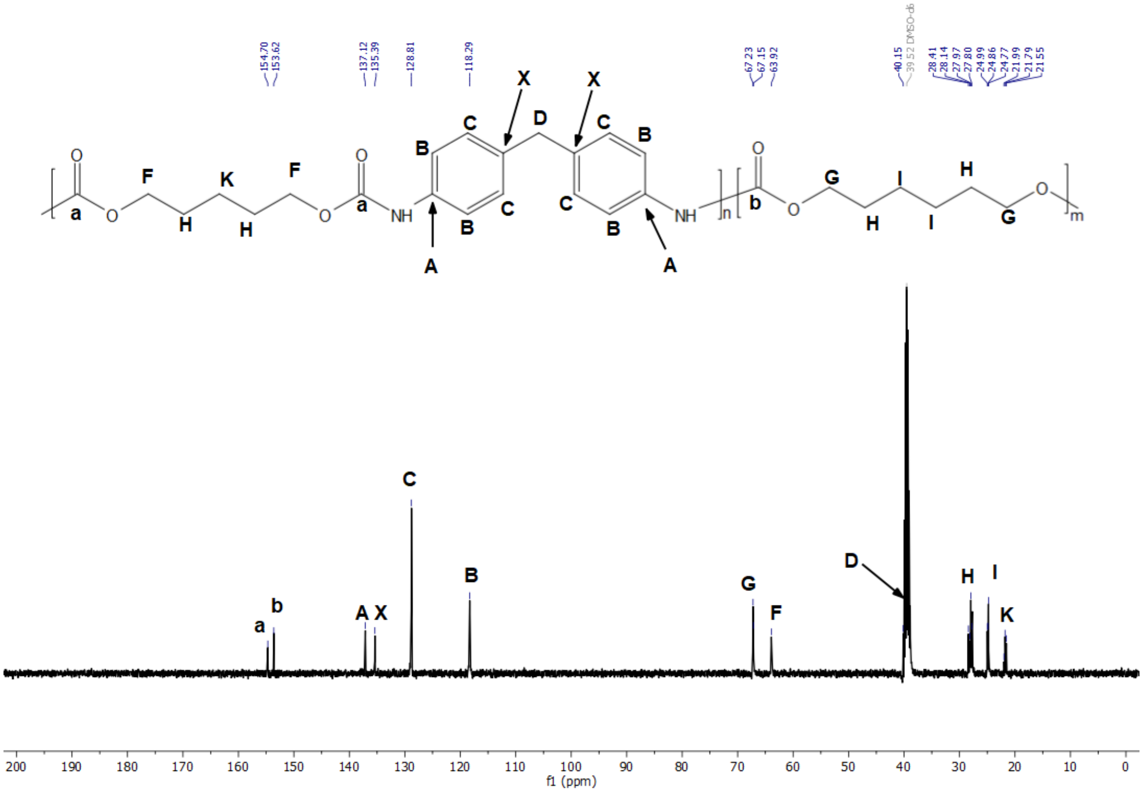

Figure S18. ^{13}C NMR spectra of NIPCU_1_5.

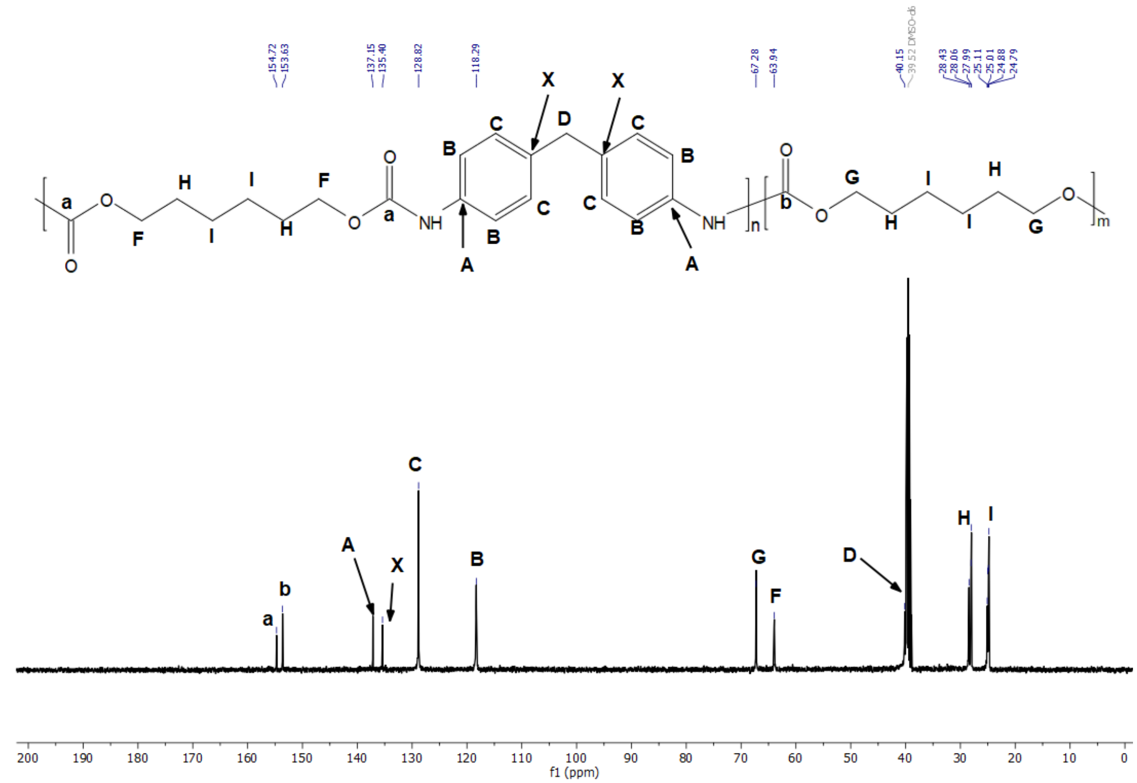

Figure S19. ^{13}C NMR spectra of NIPCU_1_6.

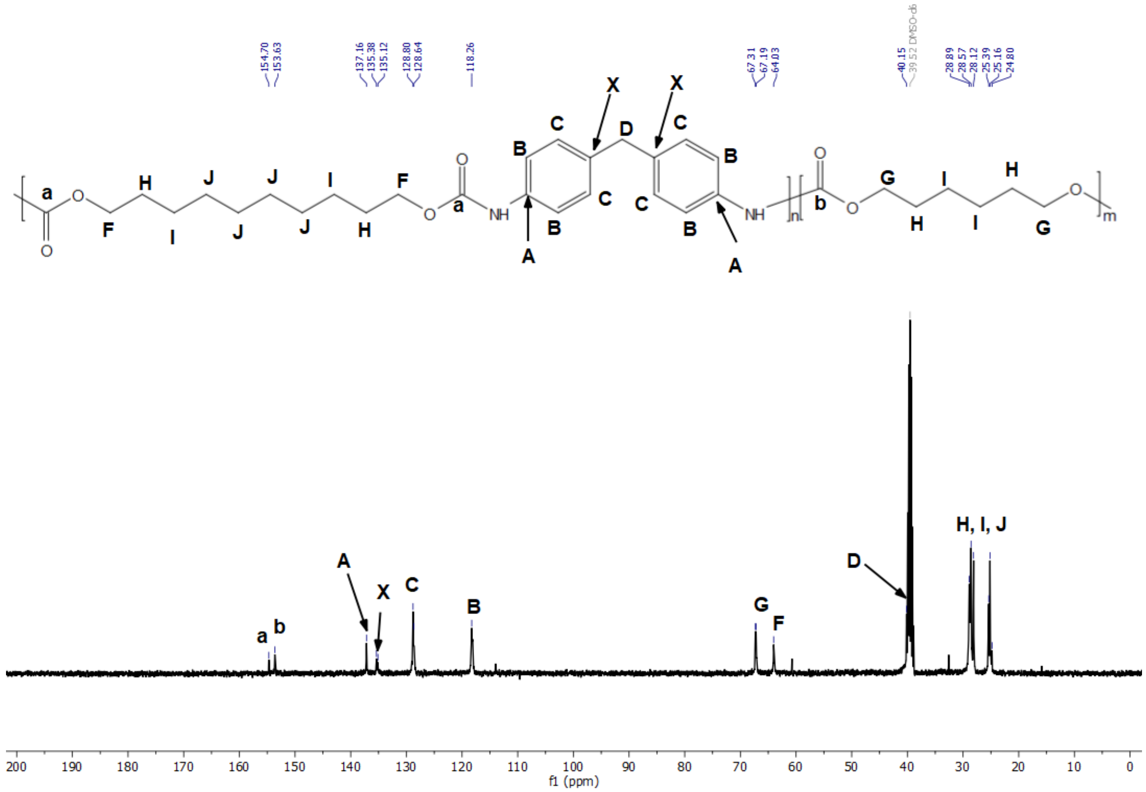

Figure S20. ^{13}C NMR spectra of NIPCU_1_10.

2.3. FT-IR spectra of NIPCUs

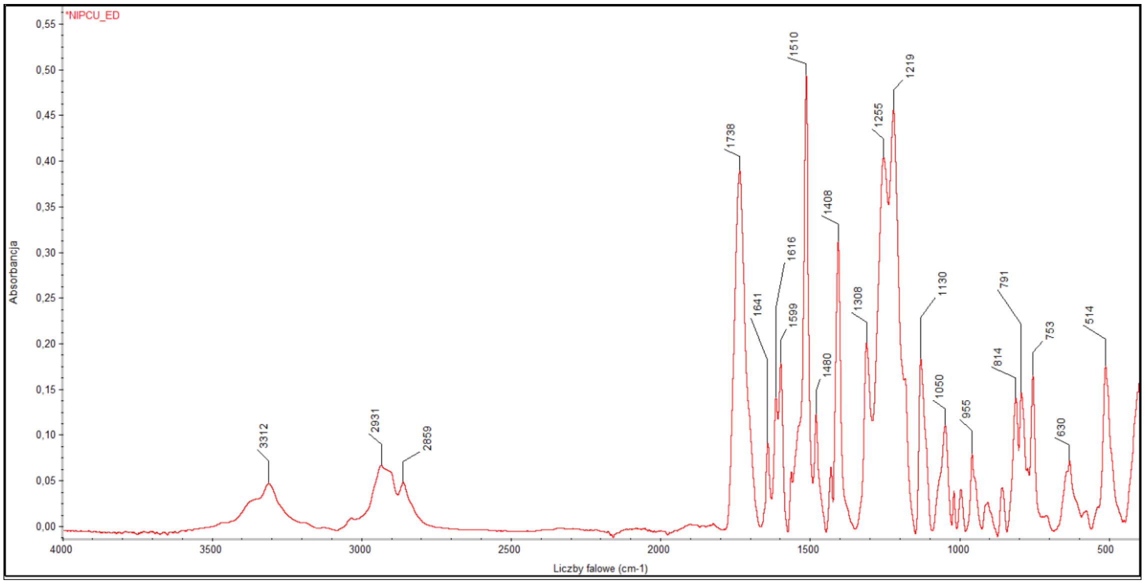

Figure S21. FT-IR spectra of NIPCU_1_2.

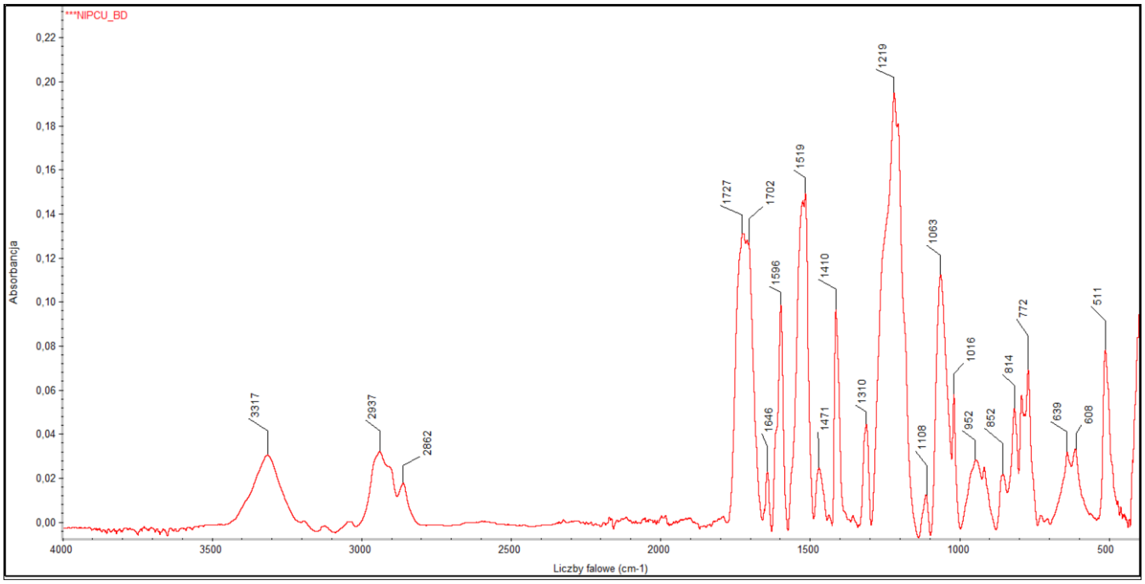

Figure S22. FT-IR spectra of NIPCU_1_4.

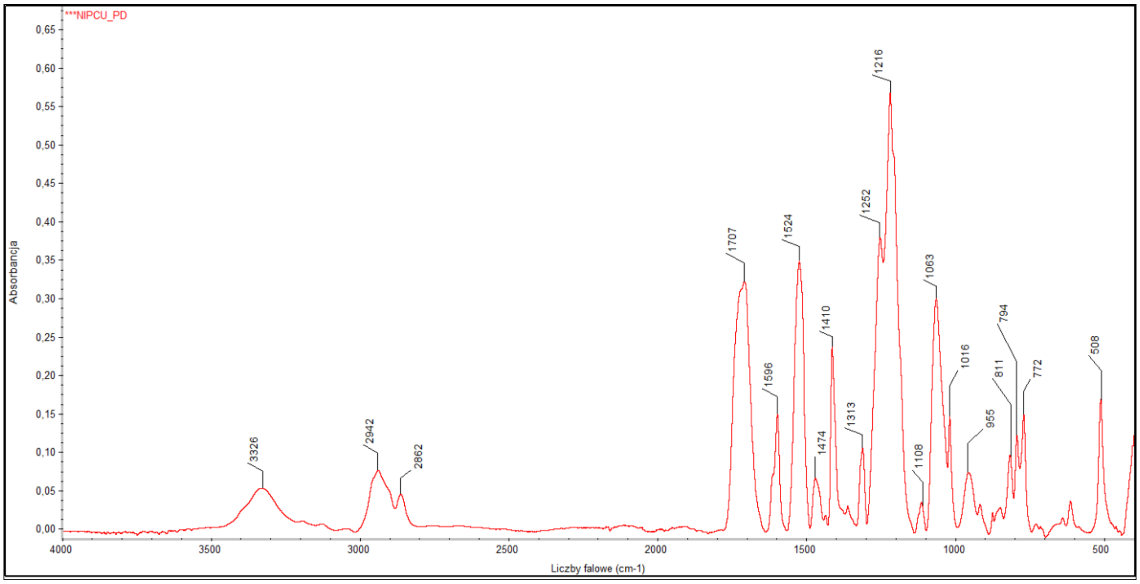

Figure S23. FT-IR spectra of NIPCU_1_5.

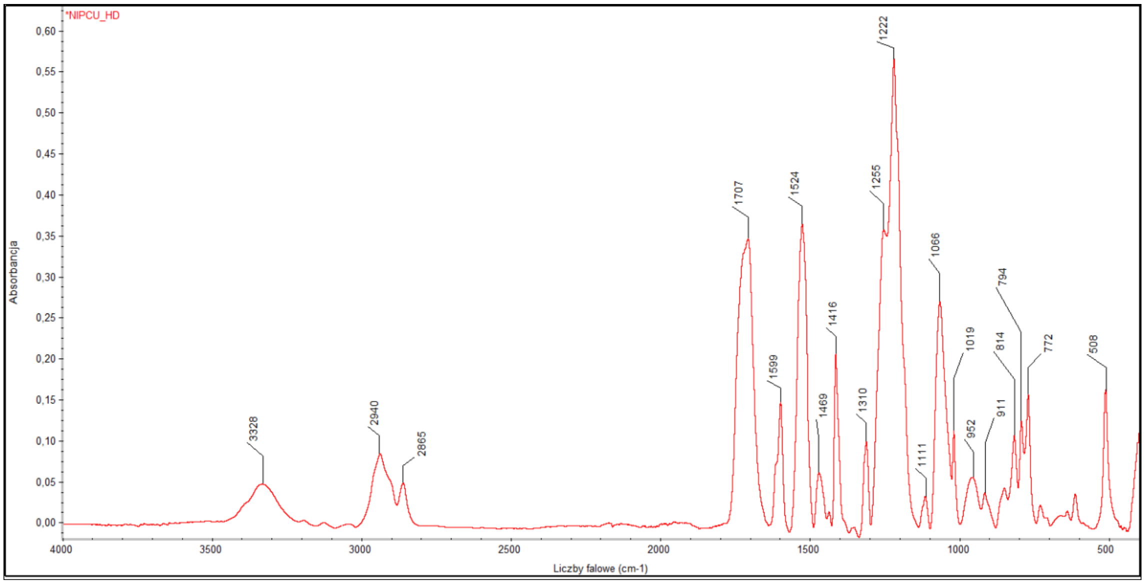

Figure S24. FT-IR spectra of NIPCU_1_6.

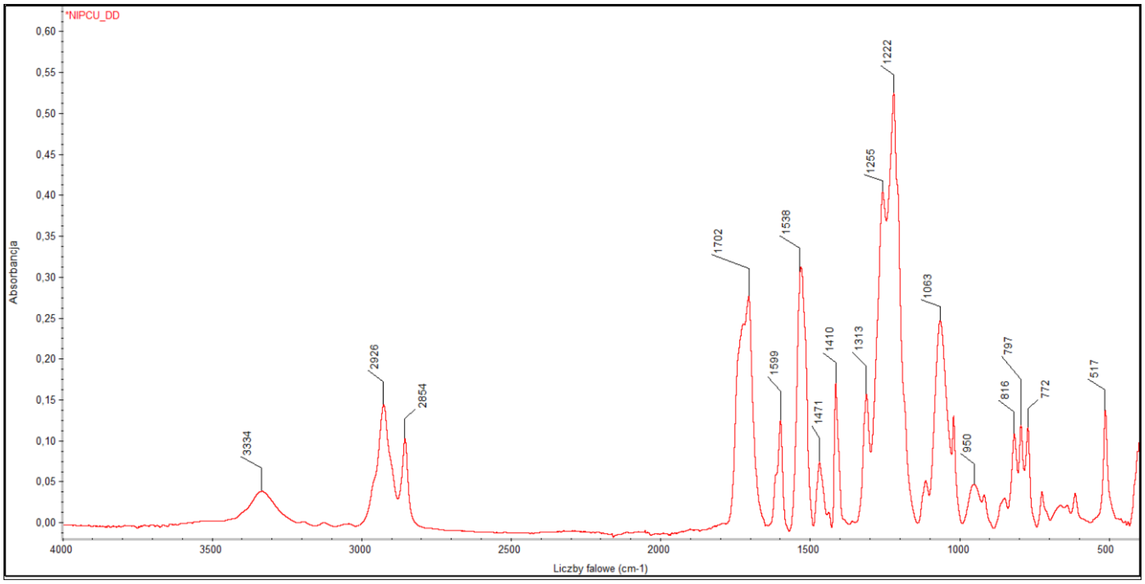

Figure S25. FT-IR spectra of NIPCU_1_10.

2.4. ^{1}H NMR spectra of NIPCUs' distillates

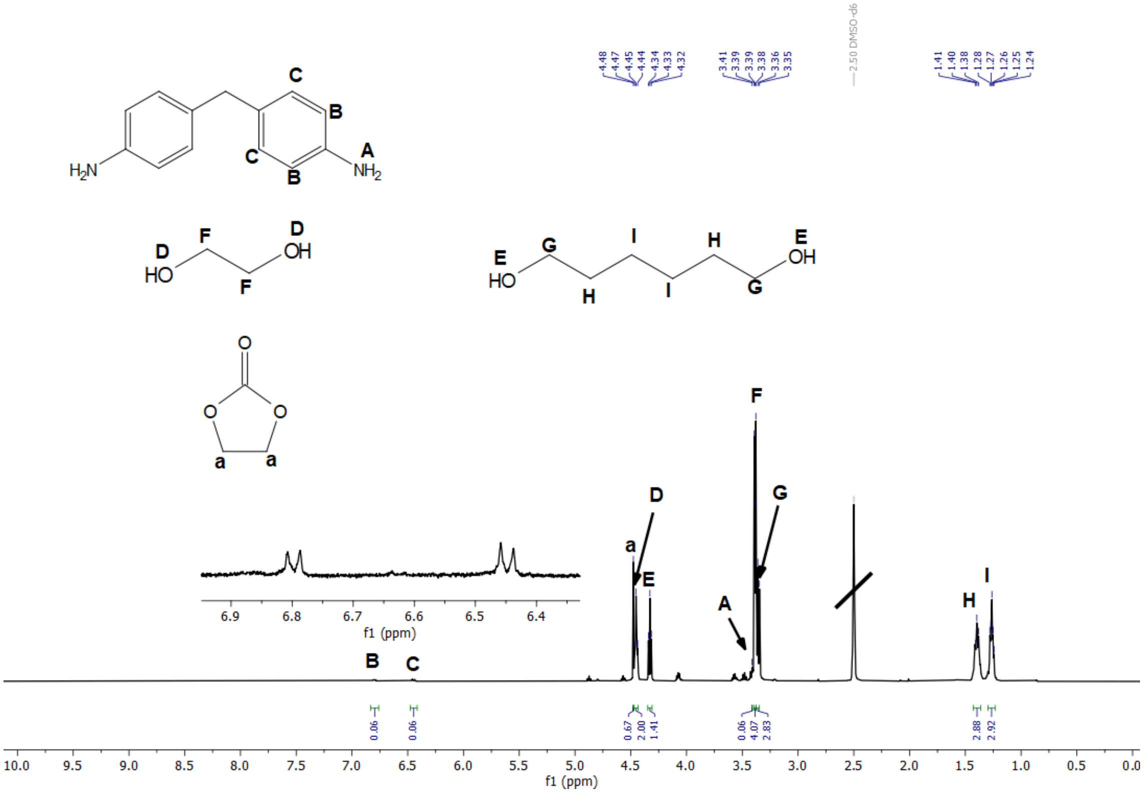

Figure S26. ^{1}H NMR spectra of NIPCU_1_2's distillate.

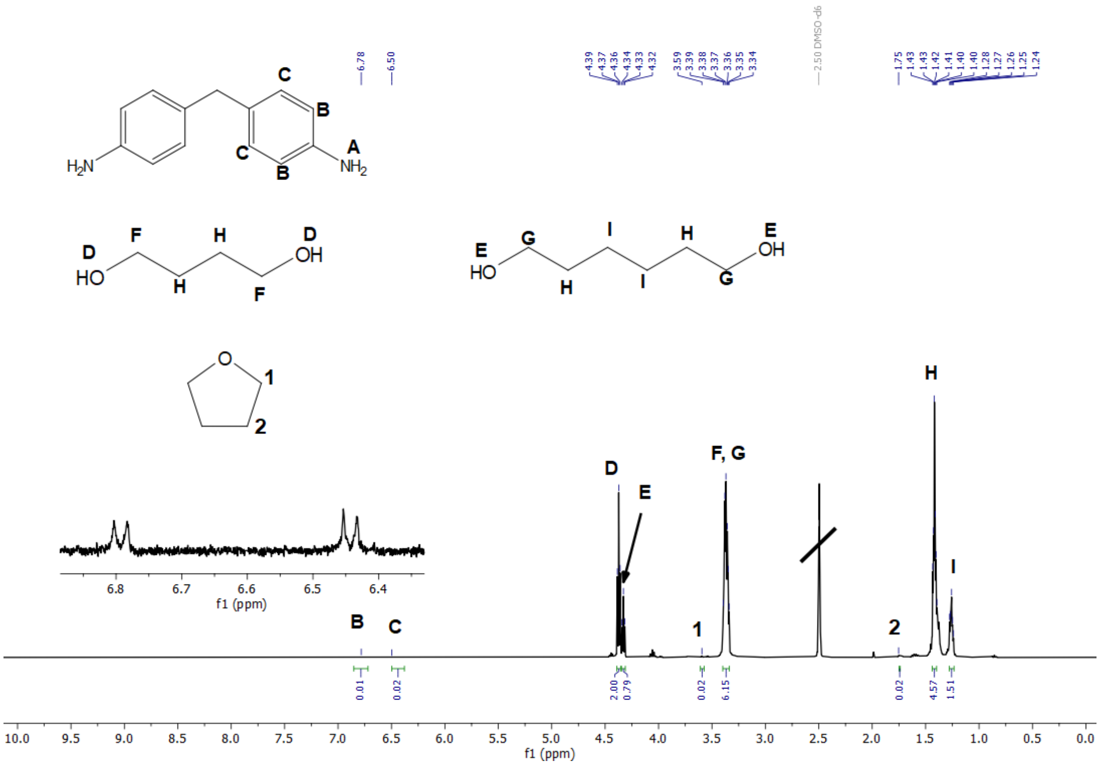

Figure S27. ^{1}H NMR spectra of NIPCU_1_4′s distillate.

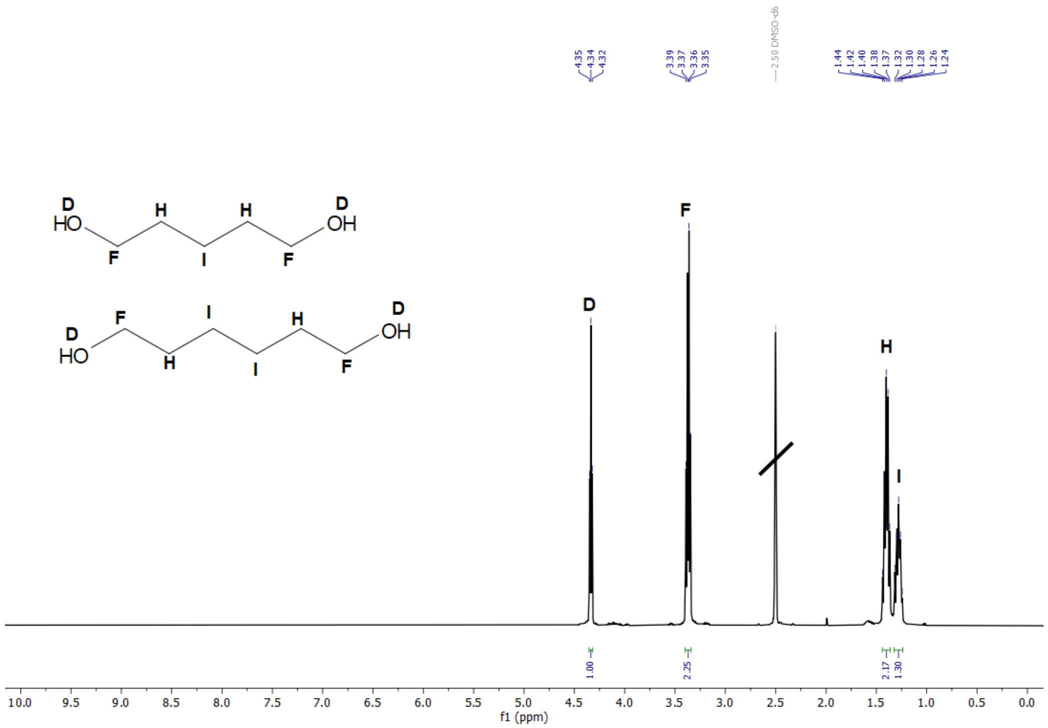

Figure S28. ^{1}H NMR spectra of NIPCU_1_5′s distillate.

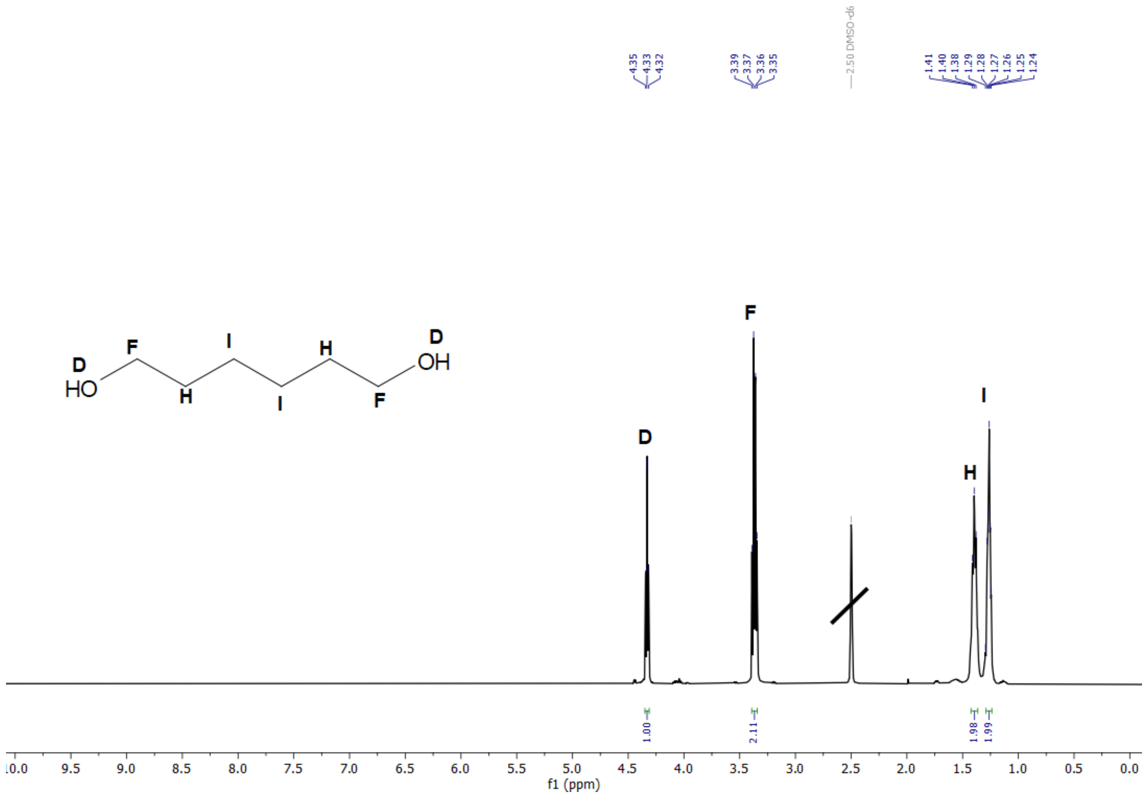

Figure S29. ^{1}H NMR spectra of NIPCU_1_6's distillate.

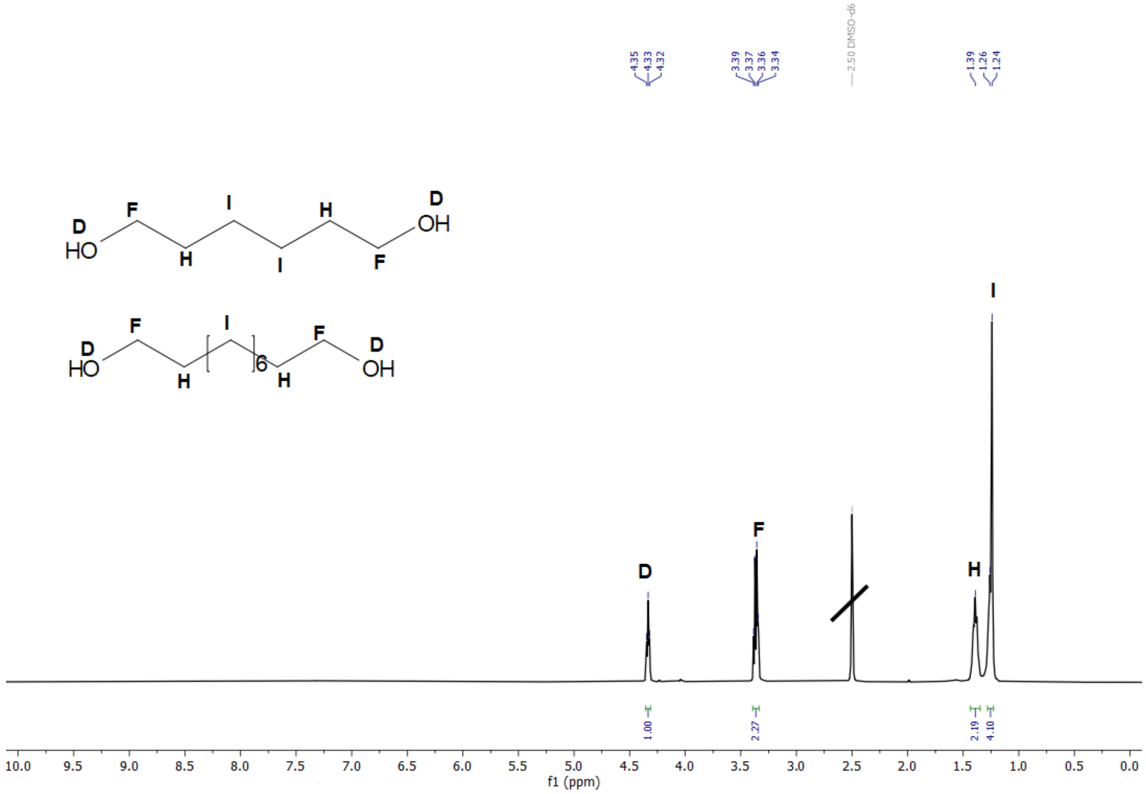

Figure S30. ^{1}H NMR spectra of NIPCU_1_10's distillate.

3. Calculations

The concentrations of urethane and carbonate groups in NIPCUs were calculated according to the Equation (S1). In case of urethane groups, the integration of the characteristic signal from the **CH_2**OC(O)NH groups (NIPCU_1_2 and NIPCU_1_10) or H_{ar} (7.35 ppm; remaining NIPCUs) were used for calculations, while in case of carbonate groups, the characteristic signal from the **CH_2**OC(O)O groups (NIPCU_1_2 and NIPCU_1_10) or CH_2 groups (1.28 ppm; remaining NIPCUs) were used.

$$\mathrm{mol}_{\%}\,\text{of urethane groups} = \frac{\frac{\text{Integral}_{\text{urethane}}}{4}}{\frac{\text{Integral}_{\text{urethane}}}{4} + \frac{\text{Integral}_{\text{carbonate}}}{4}} \qquad \text{(S1)}$$

Theoretical concentrations of urethane and carbonate groups in NIPCUs were calculated based on the concentration of functional groups in the monomers. During the transurethane polycondensation, the 90 mol% of BHAC and 10 mol% of OCD were used. The number-average molar mass of OCD was 2000 g·mol^{-1}, hence the 1 mol of OCD contained the 13.07 moles of carbonate groups. Therefore, the 180 mol of urethane groups and about 131 moles of carbonate groups were present during the transurethane polycondensation. The theoretical concentrations of urethane groups were calculated based on the Equation (S2).

$$\text{theoret. mol}_{\%}\,\text{of urethane groups} = \frac{180}{180 + 130.7} = 57.93\ mol\% \qquad \text{(S2)}$$

The same signals from hard (HS) and soft (SS) segments, as in case of the concentrations of urethane and carbonate groups, were used to calculate the number-average molar mass of the NIPCUs, according to the Equation (S3).

$$\overline{M_n} = \frac{Integral_{urethane}}{4} \cdot M_{HS} + \frac{Integral_{carbonate}}{4} \cdot M_{SS} + M_{end\ group} \qquad \text{(S3)}$$

The molar mass of the end groups and the repetitive units, in case of different HS and SS, were as follow:

NIPCU_1_2: end group = 62 g·mol^{-1}, M_{HS} = 312 g·mol^{-1}, M_{SS} = 144 g·mol^{-1};
NIPCU_1_4: end group = 90 g·mol^{-1}, M_{HS} = 340 g·mol^{-1}, M_{SS} = 144 g·mol^{-1};
NIPCU_1_5: end group = 104 g·mol^{-1}, M_{HS} = 354 g·mol^{-1}, M_{SS} = 144 g·mol^{-1};
NIPCU_1_6: end group = 118 g·mol^{-1}, M_{HS} = 368 g·mol^{-1}, M_{SS} = 144 g·mol^{-1};
NIPCU_1_10: end group = 174 g·mol^{-1}, M_{HS} = 424 g·mol^{-1}, M_{SS} = 144 g·mol^{-1}.
